# PPARγ-dependent hepatic macrophage switching acts as a central hub for hUCMSC-mediated alleviation of decompensated liver cirrhosis in rats

**DOI:** 10.1186/s13287-023-03416-2

**Published:** 2023-07-27

**Authors:** Yunqi Yao, Lin Zhang, Fuyi Cheng, Qingyuan Jiang, Yixin Ye, Yushuang Ren, Yuting He, Dongsheng Su, Lin Cheng, Gang Shi, Lei Dai, Hongxin Deng

**Affiliations:** 1 State Key Laboratory of Biotherapy and Cancer Center/Collaborative Innovation Center of Biotherapy, West China Hospital, Sichuan University, Ke-yuan Road 4, No.1, Gao-peng Street, Chengdu, 610041 People’s Republic of China; 2 Department of Obstetrics, Sichuan Provincial Hospital for Women and Children, Chengdu, People’s Republic of China; 3 Laboratory of Pathology, Key Laboratory of Transplant Engineering and Immunology, NHC, West China Hospital, Sichuan University, Chengdu, People’s Republic of China

**Keywords:** Decompensated liver cirrhosis, hUCMSCs, Stem cell therapy, Macrophages, PPARγ

## Abstract

**Background:**

Decompensated liver cirrhosis (DLC), a terminal-stage complication of liver disease, is a major cause of morbidity and mortality in patients with hepatopathies. Human umbilical cord mesenchymal stem cell (hUCMSC) therapy has emerged as a novel treatment alternative for the treatment of DLC. However, optimized therapy protocols and the associated mechanisms are not entirely understood.

**Methods:**

We constructed a DLC rat model consistent with the typical clinical characteristics combined use of PB and CCL_4_. Performing dynamic detection of liver morphology and function in rats for 11 weeks, various disease characteristics of DLC and the therapeutic effect of hUCMSCs on DLC in experimental rats were thoroughly investigated, according to ascites examination, histopathological, and related blood biochemical analyses. Flow cytometry analysis of rat liver, immunofluorescence, and RT-qPCR was performed to examine the changes in the liver immune microenvironment after hucMSCs treatment. We performed RNA-seq analysis of liver and primary macrophages and hUCMSCs co-culture system in vitro to explore possible signaling pathways. PPARγ antagonist, GW9662, and clodronate liposomes were used to inhibit PPAR activation and pre-exhaustion of macrophages in DLC rats’ livers, respectively.

**Results:**

We found that changing the two key issues, the frequency and initial phase of hUCMSCs infusion, can affect the efficacy of hUCMSCs, and the optimal hUCMSCs treatment schedule is once every week for three weeks at the early stage of DLC progression, providing the best therapeutic effect in reducing mortality and ascites, and improving liver function in DLC rats. hUCMSCs treatment skewed the macrophage phenotype from M1-type to M2-type by activating the PPARγ signaling pathway in the liver, which was approved by primary macrophages and hUCMSCs co-culture system in vitro. Both inhibition of PPARγ activation with GW9662 and pre-exhaustion of macrophages in DLC rats’ liver abolished the regulation of hUCMSCs on macrophage polarization, thus attenuating the beneficial effect of hUCMSCs treatment in DLC rats.

**Conclusions:**

These data demonstrated that the optimal hUCMSCs treatment effectively inhibits the ascites formation, prolongs survival and significantly improves liver structure and function in DLC rats through the activation of the PPARγ signaling pathway within liver macrophages. Our study compared the efficacy of different hUCMSCs infusion regimens for DLC, providing new insights on cell-based therapies for regenerative medicine.

**Supplementary Information:**

The online version contains supplementary material available at 10.1186/s13287-023-03416-2.

## Introduction

Decompensated liver cirrhosis (DLC) is the end stage of chronic liver disease, various etiologies, such as viral hepatitis and steatohepatitis induce that [1]. The prognosis of patients with DLC is relatively poor, and the estimated 5-year survival rate is 14–35% [2]. Liver transplantation is the definitive treatment for DLC; however, this strategy is limited by a shortage of available donors and several adverse effects. Therefore, effective alternative strategies for DLC therapy are urgently needed.

Cell therapy is a promising treatment for end-stage liver diseases. MSCs have attracted increasing attention for the treatment of hepatic diseases owing to their abundance, high proliferative activity, and low immunogenicity [3–5]. According to several clinical studies, MSCs therapy improves liver function and alleviates-related complications in patients with liver cirrhosis [6–11]. However, only one study found a long-term survival benefit at the 75-month follow-up after a combination of UC-MSC infusion and conventional drug therapy [11]. Moreover, other studies revealed the limited effects of MSCs and even a lack of effect on liver cirrhosis [12, 13]. In addition, the factors influencing the efficacy of MSCs have been widely studied and reported in terms of administration route and optimal dose. However, only a few studies revealed the effect of differences in MSC infusion starting time, infusion frequency, and infusion interval on the efficacy of MSCs in DLC. Therefore, addressing these issues in DLC animal models is thus of great significance for clinical application.

Compared with other common MSCs used for treating liver disease (such as adipose and bone marrow derived-MSCs), hUCMSCs have more advantages for many reasons, including potential noninvasive isolation, with higher proliferative activity, and a stronger immunomodulatory capacity [14–16]. Most importantly, hUCMSCs secrete large amounts of hepatocyte growth factor, which has been demonstrated to promote the growth of hepatocytes and inhibit liver fibrosis [17, 18]. As a result, hUCMSCs are the most favorable seed cells for treating liver disease. Based on previous studies, hUCMSCs exert therapeutic effects on experimental hepatic fibrosis and cirrhosis [19–21]. Currently, some studies show that activation of PPARγ promotes the polarization of M2-type macrophages to prevent the development of liver disease [22–24]. However, the regulatory effect of hUCMSCs on liver macrophages PPARγ is still unclear, and whether hUCMSCs infusion plays a therapeutic role by regulating PPARγ-dependent hepatic macrophage switching has not been reported. Accordingly, the optimal treatment regimens and molecular mechanisms of hUCMSCs on DLC remain obscure. An in-depth exploration of these issues is thus of positive significance for promoting basic research on hUCMSCs for clinical transformation.

In this study, we constructed a DLC rat model consistent with the typical clinical characteristics. By performing dynamic detection of liver morphology and function in rats for 11 weeks, the various stages and corresponding disease characteristics of DLC in experimental rats were fully understood. Using DLC rat models, we evaluated the influences of key therapeutic options and the therapeutic effects of hUCMSCs on DLC, which indicated that the optimal hUCMSCs treatment schedule is once every week for three weeks in the early stage of DLC progression; this schedule could significantly improve various typical characteristics of DLC rats. Mechanistically, hUCMSCs polarize M1-type macrophages to M2-type macrophages by activating the PPARγ signaling pathway in the liver macrophages of DLC rats. Collectively, this study provides a theoretical basis and treatment regimen selection for the clinical application of hUCMSCs in DLC patients, thereby serving as innovative research of significant value in clinical application.

## Materials and methods

### Isolation and identification of hUCMSCs

Human umbilical cord tissue was obtained from three healthy donors at the Sichuan Maternal and Child Health Hospital, following their consent according to procedures approved by the Medical Ethics Committee of Sichuan University (K2018109-1). HUCMSCs were isolated and purified, and their immunophenotype and differentiation potential were determined according to reported procedures [25, 26]; the results are shown in Additional file 1: Figure S1. HUCMSCs were cultured in MSC basal medium (DAKEWE, Beijing, China) supplemented with 5% UltraGROTM (HPCFDCRL50, Helios). Cells between passages 5 and 6 were used for all experiments.

### Imaging DiR-labelled hUCMSCs in vivo using the IVIS system

To monitor the biodistribution of hUCMSCs in DLC rats, hUCMSCs labeled with the fluorescent lipophilic tracer, DiR, were intravenously injected into DLC rats after four weeks of modeling. HUCMSCs were labeled according to the manufacturer’s instructions. Fluorescence imaging distribution was observed using a Small Animal Optical Imaging System (IVIS Spectrum, Perkin Elmer). The results are shown in Additional file 1: Figure S2.

### Isolation and identification of Kupffer and peritoneal macrophages

Adult male Wistar rats (approximately 200 g) were used to obtain rat Kupffer cells and peritoneal macrophages; the detailed isolation methods were according to the reported protocols [27, 28]. The primary cells were cultured in MaM medium (basal medium + 5% FBS + 1% MaGS + 1% P/S). All cells were cultured in a 37 °C, 5% CO_2_ incubator.

### HUCMSCs and macrophages were co-cultured in Transwell

In vitro co-culture experiments, hUCMSCs were inoculated in the upper chamber of Transwell and cultured with MSC basal medium. Macrophages were seeded in a lower chamber and cultured with MaM medium. The upper and lower cultures were separated by polycarbonate membranes. Due to the permeability of the membranes, the components secreted by hUCMSCs in the upper cultures could affect the macrophages in the lower chamber. After 24 h of co-culture, the macrophage phenotype was observed.

### DLC rat model and hUCMSCs treatment

Male Wistar rats were purchased from Beijing Huafu-Kang Biotechnology Co., Ltd. (China). The procedure for using the animals followed the Guidance Suggestions for the Care and Use of Laboratory Animals formulated by the Ministry of Science and Technology of China. All experimental procedures involving animals were approved by the Sichuan University Medical Ethics Committee (K2018109-2). To induce decompensated liver cirrhosis model, 8-week-old male Wistar rats (220 ± 30 g) were intraperitoneally injected with CCl_4_ (0.5 mL/kg body weight, dissolved in olive oil, 1:1; Sigma-Aldrich) twice per week for 11 weeks, and at the same time, with 0.35 g/L phenobarbital (Sigma-Aldrich) in the rats’ drinking water. To collect rat blood and liver samples, rats were anesthetized by intramuscular injection of Zoletil®50 (zolazepam–tiletamine) at a dosage of 100μL/100 g according to the body weight. The use of anesthetic Zoletil®50 does not cause any pain to the rats, in compliance with animal ethics. After the sampling was completed, euthanasia of the rats was carried out under anesthesia using cervical dislocation method. The typical features of decompensated cirrhosis, including ascites, impaired liver function, and high mortality, were observed at the end of week 11; the results are shown in Additional file 1: Figure S3. According to our experimental design, hucMSCs (6 × 10^6^ cells/kg) were administered intravenously once per week or three times per week for three consecutive weeks at the 5th and 8th of modeling, respectively. Samples were harvested at the end of 11 weeks of modeling to evaluate the treatment efficacy of hucMSCs.

### Abdominal ultrasound and imaging acquisition

All rats underwent abdominal ultrasonography once per week during DLC modeling using a PHILIPS real-time ultrasonography with a low-frequency convex array probe at 42 Hz.

### Examination of rats’ blood biochemical index

The venous blood of rats was placed in procoagulant tubes or anticoagulant tubes and then centrifuged at 1800 rpm for 15 min at 4 °C to obtain serum and plasma, respectively. The activities of serum alanine aminotransferase (ALT), aspartate aminotransferase (AST), lactate dehydrogenase (LDH), Alkaline phosphatase (ALP), albumin (ALB), total bilirubin (TBIL), gamma-glutamyl transpeptidase (GGT), and creatinine (CREA) were determined at GLP workshop of the National New Drug Safety Evaluation Center (WestChina-Frontier PharmaTech).

### Cytokine detection using the Luminex assay

Rat serum cytokines were detected using multifactor Luminex Assay according to the manufacturer’s instructions (Luminex, Texas, America). First, the antibody arrays were incubated with the blocking buffer at room temperature for 30 min. The serum sample diluted in blocking buffer was added to the corresponding well at 4 °C overnight. The wells were rinsed three times with wash buffer I (5 min each time) and then two times with wash buffer II at room temperature (10 min each time). The antibody arrays were incubated with the biotinylated antibody cocktail diluted in the blocking buffer at room temperature for 2 h and washed as mentioned above; the HRP-anti protein-streptomycin was diluted with blocking buffer, added to the wells, incubated at room temperature for 2 h, and then washed as mentioned above; after mixing the detection buffer C and detection buffer D at a ratio of 1:1, the mixture was incubated with the antibody arrays for 2 min. Finally, the signal was read using the Luminex instrument.

### Flow cytometry

Animals were euthanized at the end of week 11. The livers were harvested, minced, and digested in RPMI-1640 medium containing collagenase IV (0.1%; Gibco), nuclease, and 1% fetal bovine serum (FBS) at 37 °C for 40–60 min, and the cell suspensions were filtered. Fixable viability stain 620 (BD Biosciences) was used to discriminate between live and dead cells. Finally, the cells were blocked with Fc-Block (BD Biosciences) and stained with antibodies. The data were acquired using a NovoCyte flow cytometer.

### Histological analysis and immunohistochemistry

Liver tissues were harvested, immediately fixed with 4% paraformaldehyde, and embedded in paraffin for subsequent use. Deparaffinized liver sections were sectioned at 4-μm thickness for liver histology and stained with red hematoxylin and eosin (H&E). Deparaffinized liver sections were subjected to citric acid buffer (PH6.0) microwave antigen retrieval for immunohistochemistry and then treated with 0.3% H_2_O_2_ solution to block endogenous peroxidase. After washing, the sections were blocked with non-immune serum and incubated overnight with primary antibodies at 4 °C. These sections were then incubated with a chromogenic reagent until the liver sections turned brown. Four-to-eight independent liver sections were randomly collected using Nikon ECLIPSE E600, and the number of positive cells was quantified using ImageJ software.

### Immunofluorescence

Liver tissues were harvested, fixed with 4% paraformaldehyde for 24 h, dehydrated with 30% sucrose, and embedded in the OCT compound. Briefly, 4-µm-thick frozen liver sections were incubated with primary antibody at 4 °C overnight after antigen recovery. The secondary antibody was subsequently added for 1 h at room temperature. Nuclei were stained with Hoechst 33,258 at room temperature for 10 min. Frozen sections were observed and photographed using a fluorescent microscope (Leica, Germany). Four-to-eight independent liver sections were randomly collected, and the number of positive cells was quantified using ImageJ software.

### Real-time PCR

Total RNA was extracted using TRIzol reagent (Life Technologies, Carlsbad, CA, USA). After the concentration was measured, RNA was reverse-transcribed, and mRNA expression analysis was performed using the PrimeScript RT Reagent Kit (TaKaRa, Japan) on a LightCycler 96 System (Roche, Basel, Switzerland). Gene expression was normalized to that of the housekeeping gene, β-actin. The primers used are listed in Additional file 1: Table S1.

### RNA-seq

Total RNA from the liver tissues was isolated as described above. RNA sequencing was performed by Chengdu Basebiotech Co., Ltd (Chengdu, China). In short, RNA purity was assessed using the AMPure XP system (Beckman Coulter, Beverly, USA). Sequencing libraries were prepared using the NEBNext® UltraTM RNA Library Prep Kit (Illumina, NEB, USA) according to the manufacturer’s protocol. Samples were ligated to unique adaptors and subjected to PCR amplification. Libraries were validated using an Agilent Bioanalyzer 2100, normalized, and pooled for sequencing. RNA-seq libraries prepared from at least three biological replicates for each group were sequenced on an Illumina Novaseq using barcoded multiplexing and a 150-bp read length. The raw data were normalized using DESeq2. All RNA-seq raw data analyzed in this study are deposited in the NCBI SRA database (Accession No. SRP287012).

### Western blotting

Proteins were extracted from the treated cells or tissues, as indicated, using RIPA lysis buffer (Beyotime, Nanjing, China) containing a 1% protease inhibitor cocktail (Merck Millipore, Birrika, USA), and prepared for SDS-PAGE loading buffer (Abclonal, Wuhan, China). Proteins were separated via sodium dodecyl sulfate–polyacrylamide gel electrophoresis using 10% Tris–glycine mini-gels and transferred onto a PVDF membrane (0.45 μm, Immobilon-P Transfer Membranes, Merck Millipore). The primary antibodies used are listed in Additional file 1: Table S2, and the corresponding dilutions are 1:1000 according to the antibody instructions. GAPDH mAb (Santa Cruz, Biotechnology) was used as the loading control for all experiments. Following incubation with horseradish peroxidase-conjugated secondary antibody (Zsbio, Beijing, China) for 2 h at room temperature, the bands were then tested by a chemiluminescent substrate ECL kit (Yamei, Shanghai, China).

### Statistical analysis

Data were analyzed using Prism software (GraphPad Prism version 5). Statistical significance was analyzed using the two-tailed Student’s test. Animal survival was presented using Kaplan–Meier survival curves and analyzed using the log-rank test. Differences were considered statistically significant at *p* < 0.05. The symbols used to denote significance are as follows: *p < 0.05, **p < 0.01, ***p < 0.001, ****p < 0.0001, and ns (no statistical significance).

## Results

### Establishment and evaluation of the DLC rat model

To evaluate the effect of hUCMSCs-infusion on DLC, a rat model that largely conforms to the typical clinical features of DLC was established, according to published research. Based on histopathological examination, the combined use of PB and CCL_4_ could induce necrosis and the infiltration of inflammatory cells in rat liver. At week 2, the fibroid tissue in the hepatic portal area was found to gradually increase, and liver fibrosis was observed (Additional file 1: Figure S4A and B). After four weeks of drug treatment, pseudo-lobular-like structures were found in the liver of most DLC rats, and they were accompanied by ascites formation (Additional file 1: Figure S4D), one of the most typical features of decompensated cirrhosis, indicating that 4-week cumulative drug treatment could effectively promote the formation of DLC in rats. After continued treatment for seven weeks, the liver structure of DLC rats was destroyed. Consistently, extensive hepatocyte necrosis, numerous inflammatory cell infiltrations, and the formation of massive ascites were observed (Additional file 1: Figure S4A–E). Additionally, the hydroxyproline content in the liver of model rats increased linearly during the modeling period and reached a high value at 8–11 weeks (Additional file 1: Fig S4F). Based on the examination of plasma prothrombin time (PT) and serum albumin (ALB), total bilirubin (TBIL), and the content of creatinine (CREA), the results suggest that the liver function of the model rats was most severely damaged at 8–11 weeks after modeling (Additional file 1: Figure S4G). According to the changes in liver structure, the degree of liver fibrosis, liver function, and ascites in rats, 5–7 weeks of rat modeling can be considered to correspond to the early formation stage of DLC in patients, while 8–11 weeks of rat modeling can be considered to correspond to the end stage of DLC in patients.

### Optimizing the infusion regimen is essential to the hUCMSC-based therapy for DLC

**Fig. 1:**
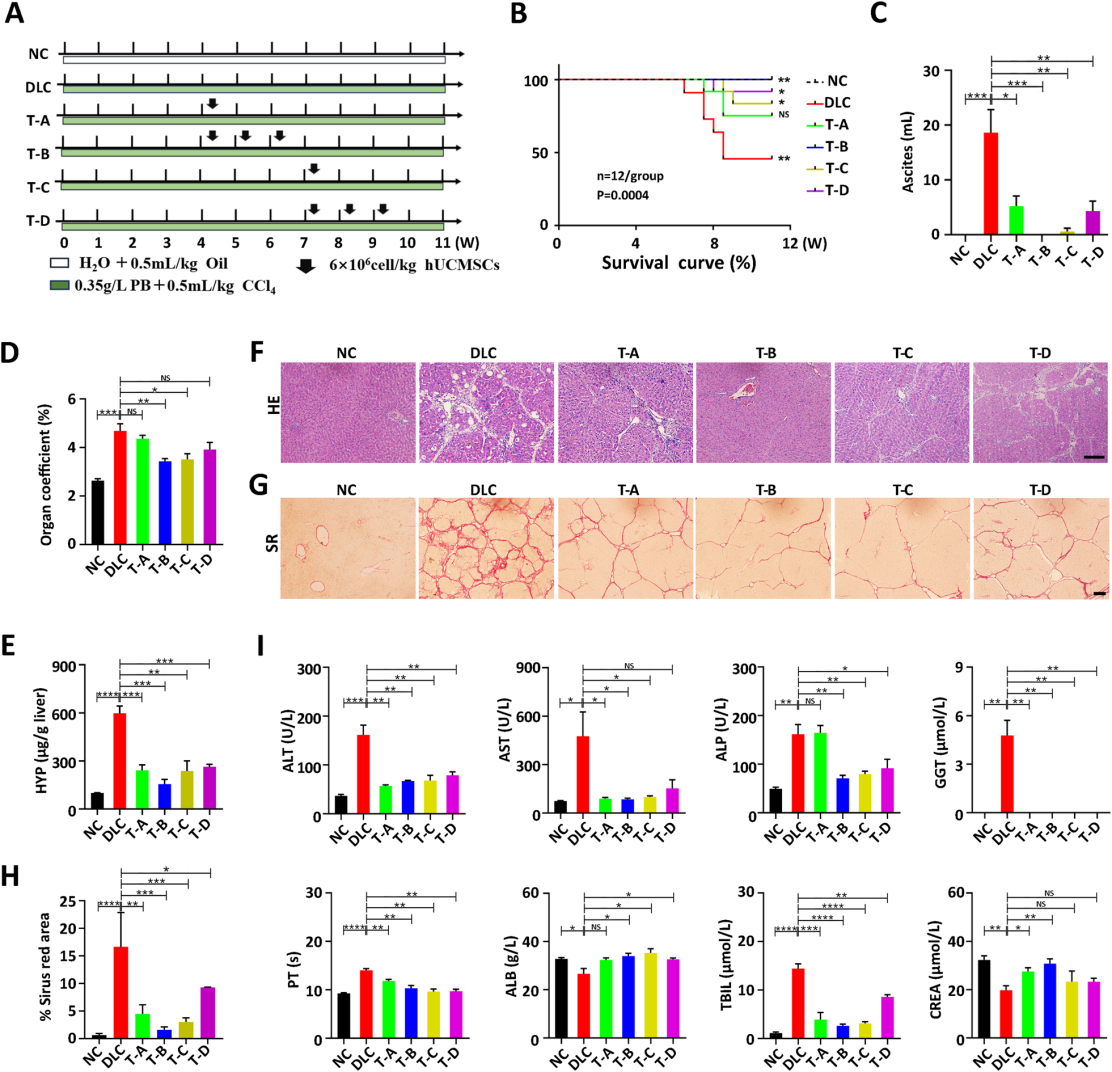
Therapeutic efficacy of four different hUCMSCs infusion regimens on DLC rats. **A** Diagram of different hUCMSCs infusion regimens for DLC rats. NC: Negative control; DLC: Decompensated liver cirrhosis rats model; T-A: A single hUCMSCs infusion at week 5 of modeling in DLC rats; T-B: Triple hUCMSCs infusion (once a week) at week 5 of modeling in DLC rats; T-C: A single hUCMSCs infusion at week 8 of modeling in DLC rats; T-D: Triple hUCMSCs infusion (once a week) at week 8 of modeling in DLC rats; **B** The survival of rats was recorded from week 6 to the end of week 11(*n* = 12). **C** The ascites was measured at the end of week 11(*n* = 5). **D** Ratio of the liver weight to the body weight in rats at the end of week 11(*n* = 4). **E** Hydroxyproline content of liver tissues following different treatment regimens was measured by Commercial kit at the end of week 11(*n* = 4). **F** and **G** Hematoxylin and eosin (H&E) and Sirius red staining of liver sections (Bar = 100 μm). **H** The quantification analysis of Sirius red positive area by ImageJ (*n* = 3). Hematoxylin and eosin (H&E) and Sirius red staining of liver sections (Bar = 100 μm). **I** Serum levels of key enzymes related to liver function at the end of week 11(*n* = 4). Data are presented as mean ± SEM. *p < 0.05, **p < 0.01, ***p < 0.001, ****p < 0.0001, and ns (no statistical significance) (all *p* values were obtained by the Two-tailed Student’s Test)

Adjusting key factors, such as the frequency of MSCs infusion and the time selection for MSCs infusion, and optimizing the treatment regimen will further improve the therapeutic effect of MSCs. As shown in the schematic diagram (Fig. 1A), the hUCMSCs infusion was performed in the early (at week 5 of modeling) and end stages (at week 8 of modeling) in DLC rats and each group was administered two treatment regimens: single and triple infusions (once a week). The four treatment regimens were labeled T-A, T-B, T-C, and T-D.

After 11 weeks of modeling, only half of the rats in the DLC group survived. In contrast, the survivability of rats in all hUCMSCs-treated groups was improved, especially the T-B group, with a survival rate of 100% (Fig. 1B). Furthermore, the level of ascites, the most distinctive feature of decompensated cirrhosis, was significantly reduced in all hUCMSCs treatment groups. In particular, ascites development was completely prevented in the T-B group (Fig. 1C). Evidently, sclerosis of the liver was significantly reduced in the T-B and T-C groups according to the liver organ coefficients (Fig. 1D). Based on the HE staining results, hUCMSC treatments reduced the inflammatory infiltration in the liver and restored the damaged liver structure (Fig. 1F). The level of hydroxyproline, the main component in collagen tissue, was also reduced in all hUCMSC treatment groups (Fig. 1E), aligning with the Sirius red staining results (Fig. 1G), which was further identified by quantification of Sirius red positive area (Fig. 1H). Notably, the most significant decrease was observed in the T-B group. The four hUCMSC treatment groups did not display a uniform response to liver function compared to the DLC group; however, the T-B group showed significant improvements in all indexes, including ALT, AST, ALP, PT, and TBIL levels, and meanwhile, the ALB and CREA levels increased (Fig. 1I). To further explore the effect of the infusion interval on hUCMSCs therapy, weekly and biweekly infusions of hUCMSCs were compared, as shown in Additional file 1: Figure S5A. Surprisingly, changes in hUCMSCs infusion intervals had no significant effects on DLC rat therapy (Additional file 1: Figure S5B-L). Altogether, these results confirm the potential therapeutic effect of hUCMSCs on DLC. More interestingly, performing hUCMSCs-based treatment at the early stage of DLC, with triple hUCMSCs infusion, could produce the best therapeutic effects, thereby providing a great reference for future basic research and the formulation of clinical treatment regimens.

### HUCMSCs improve DLC by modulating the immune microenvironment in rats, especially by shifting intrahepatic macrophages from M1 to the M2 type

**Fig. 2:**
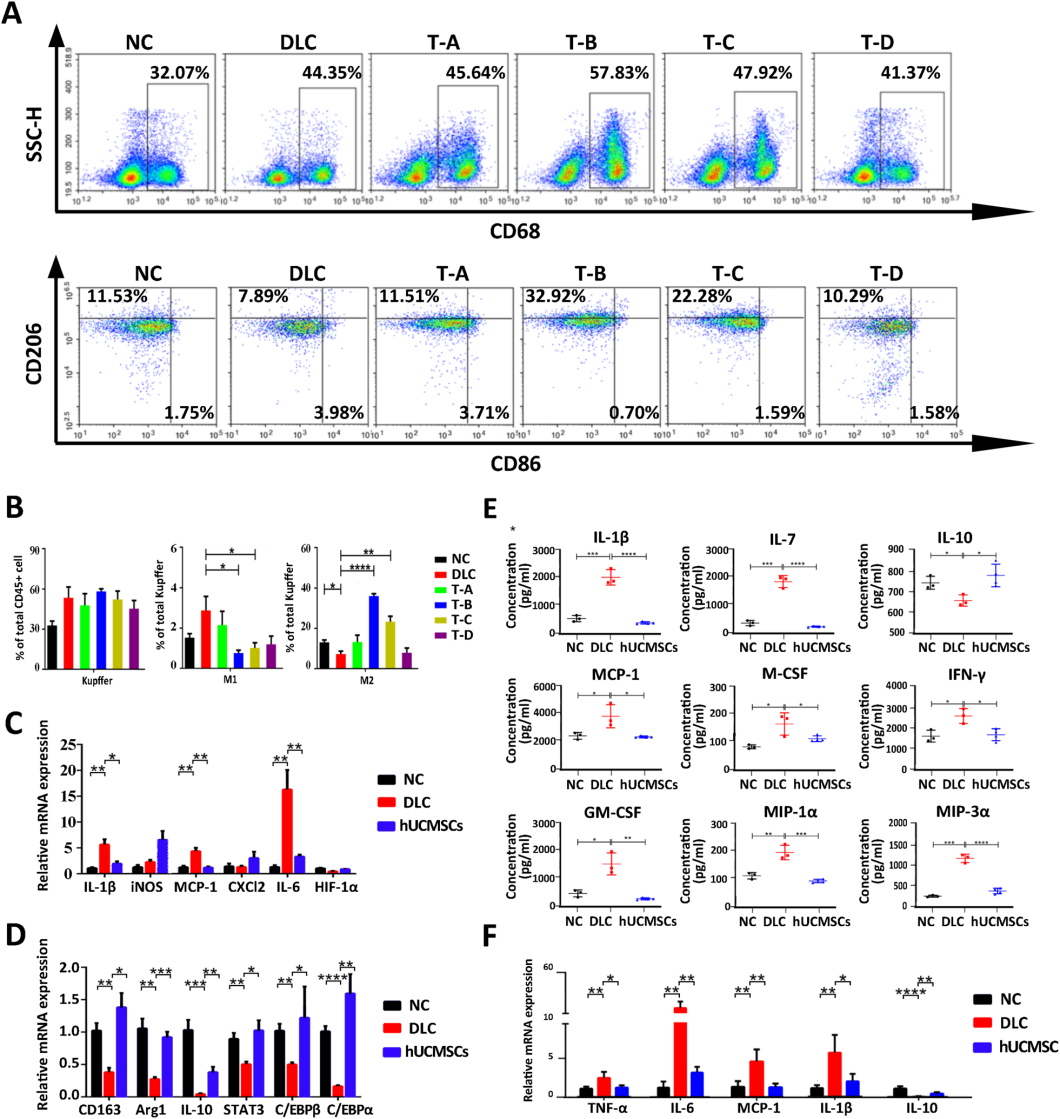
Effects of different hUCMSCs infusion regimens on the immune microenvironment in rat liver. **A** Flow cytometry analysis of the changing proportion of macrophages and their subtypes in liver tissues. **B** Percentage of macrophages and their subtypes in CD45 + T cells (*n* = 5). **C** The mRNA expression levels of M1 macrophage-related genes in the liver tissue (*n* = 5). **D** The mRNA expression levels of M2 macrophage-related genes in the liver tissue (*n* = 5). **E** The serum levels of inflammatory factors in rats based on the inflammation antibody array (NC and DLC group, *n* = 3; hUCMSCs group, *n* = 4). **F** Effect of hUCMSCs transplantation on the immune-related factors in liver tissues (*n* = 5). Data are presented as mean ± SEM. *p < 0.05, **p < 0.01, ***p < 0.001, ****p < 0.0001, and ns (no statistical significance) (all *p* values were obtained by the two-tailed Student’s test)

Based on the above findings, different hUCMSCs infusion regimens are essential for hUCMSCs therapy in DLC rats. To determine the cause of this difference, a flow cytometry analysis of the liver immune cells of DLC rats treated with hUCMSCs was performed. The proportion of total T cells among CD45+ cells was significantly increased by hUCMSCs treatment, whereas the proportions of B cells among CD45+ cells remained unchanged (Additional file 1: Figure S6A). Further analysis of the ratio of CD4+ /CD8+ T cells demonstrated that there was a significantly decreased only in the T-B group but no change in the other treatment groups (Additional file 1: Figure S6A). The percentage of neutrophils was significantly decreased in all hUCMSCs treatment groups compared with the DLC group. However, there was no significant change in the proportion of monocytes and their subpopulations among CD45+ cells (Additional file 1: Figure S6B). Additionally, considering the leading role of macrophages in the immune regulation of liver diseases, we determined the proportion of total macrophages and their subtypes, M1 and M2 macrophages. Flow cytometric analysis revealed no difference in the percentage of total macrophages among all groups, whereas the proportion of M1 macrophages in total macrophages decreased, and the proportion of M2 macrophages increased significantly in the T-B and T-C hUCMSCs treatment groups compared with the DLC group (Fig. 2A and B). To determine the effect of hUCMSCs treatment on the changes in M1 macrophages and M2 macrophages, the T-B group with the most obvious changes in M1/M2 macrophages was selected for subsequent studies. RNA expression analysis of the liver tissue from the T-B group showed that the expression levels of M1-related genes, such as IL-6, MCP-1, and IL-1β, were up-regulated in DLC rats compared with normal rats. However, the levels of these genes were significantly downregulated after hUCMSCs treatment (Fig. 2C). In contrast, the expression levels of CD163, Arg1, IL10, and other M2-related genes were significantly downregulated in the DLC group compared with the NC group and significantly up-regulated in the hUCMSCs group (Fig. 2D). To further determine whether M2 macrophages polarization can improve systemic inflammatory levels, the expression of immune-related factors in the serum of rats was examined using an Inflammation Antibody Array. The serum levels of various pro-inflammatory factors, such as IL-1β, IL-7, M-CSF, GM-CSF, and IFN-γ, were significantly decreased after hUCMSCs treatment, while the expression level of the anti-inflammatory factor, IL-10, was significantly up-regulated (Fig. 2E), which was further confirmed by examining the mRNA expression levels of inflammatory factors in the liver tissue (Fig. 2F). Although the exact molecular mechanism between different infusion regimens remains unclear, our results revealed that the change in M1/M2 macrophages proportion plays a decisive role in different infusion regimens, and further research also proved that optimal hUCMSCs infusion treatment promotes the expression of M2-related genes while inhibiting the expression of M1-related genes.

### HUCMSCs significantly increase PPARγ in the liver of DLC rats

**Fig. 3:**
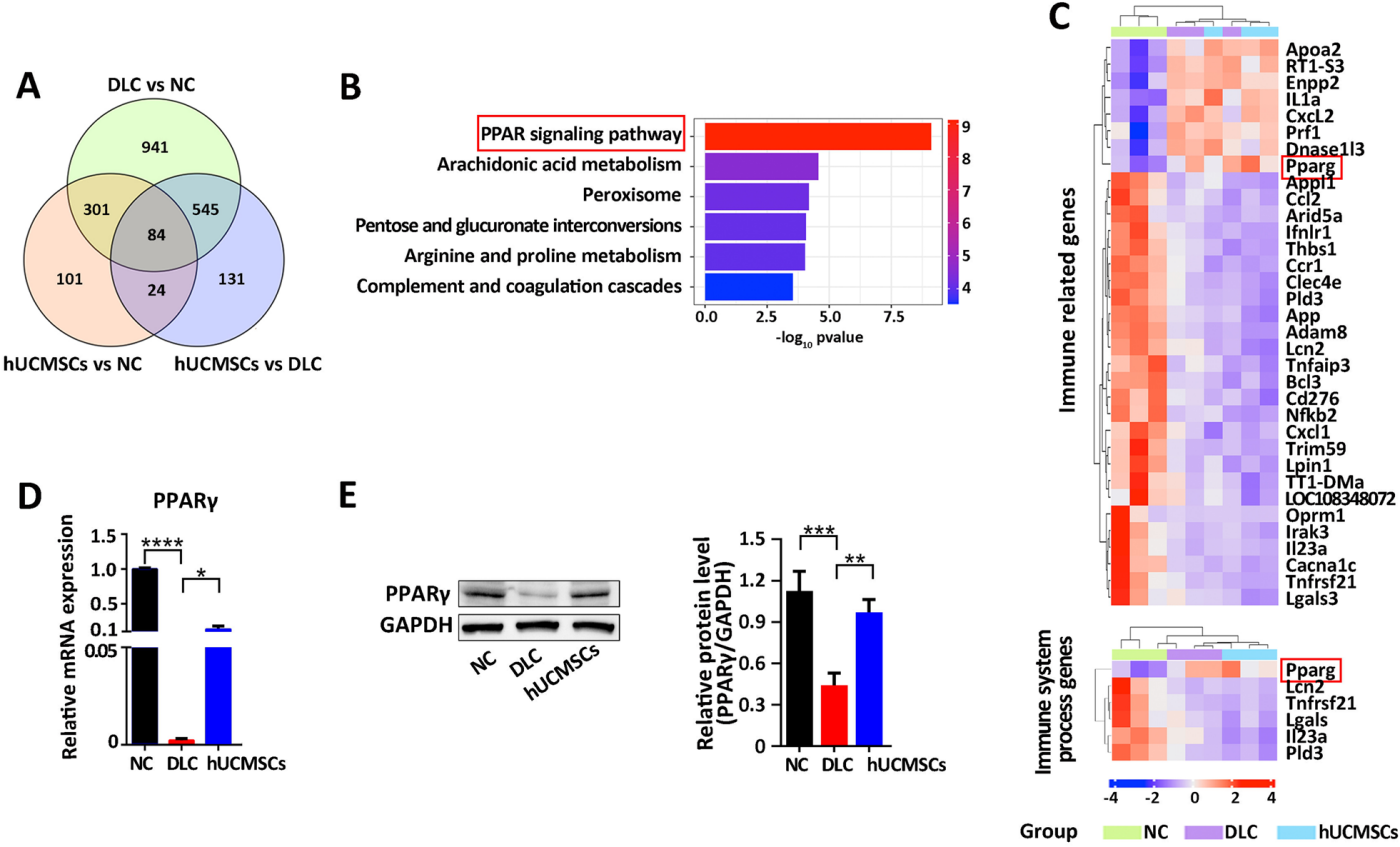
Upregulation of PPARγ in hUCMSC-treated DLC liver. **A** Venn analysis of the differential genes among the NC, DLC, and hUCMSCs groups. **B** KEGG pathways enrichment analysis of the selected differentially expressed genes. **C** Heat map of the immune-related genes among the differentially expressed genes. **D** To ascertain the results from RNA-seq, the mRNA levels of PPARγ expression were evaluated by qRT-PCR analysis (*n* = 5). **E** Protein levels of PPARγ in liver tissue (*n* = 4) (Full-length blots and repeated experiments are presented in Additional file 2). Data are presented as mean ± SEM. *p < 0.05, **p < 0.01, ***p < 0.001, ****p < 0.0001, and ns (no statistical significance) (all *p* values were obtained by the two-tailed Student’s test)

To further investigate the genomic changes in the liver of DLC rats treated with hUCMSCs, transcriptome sequencing (RNA-SEQ) of tissue samples from the normal control group (NC), decompensated cirrhosis group (DLC), and hUCMSCs treated group (hUCMSCs) was performed at the end of 11 weeks. Principal component analysis (PCA) suggested that samples in the same group had good uniformity, and samples in the hUCMSCs group were closer to those in the NC group than in the DLC group (Additional file 1: Figure S7). By performing a Venn analysis, 1871 differentially expressed genes in the DLC group were compared with the NC group, and 784 differentially expressed genes were identified in the MSC group compared with the DLC group. By comparing the NC group with the MSC group, 510 differentially expressed genes were identified; such finding aligns with the conclusion that the liver gene expression profiles of DLC rats treated with hUCMSCs were closer to those of normal rats (Fig. 3A). For the selected differentially expressed mRNAs (545 + 85), KEGG pathways enrichment analysis revealed that the gene set was mainly involved in processes related to the PPAR signaling pathway and arachidonic acid metabolism (Fig. 3B). Furthermore, heat map analysis of the immune-related genes and immune-process-related genes revealed that Cxcl1, Cxcl12, Ccr1, IL1α, IL23a, PPARγ, Ln2, and other immune-related genes in the liver of DLC rats treated with hUCMSCs were significantly different from those in the DLC group and tended to be reversed into the normal group (Fig. 3C), which illustrates that these immune factors play an important role in the development of inflammation. To further validate the RNA-seq results, RT-qPCR was performed to detect the expression of PPARγ in liver tissues. The expression of PPARγ in the DLC group was found to be significantly lower than that in the NC group. In contrast, the expression of PPARγ in the hUCMSCs group was up-regulated significantly compared with that in the DLC group, aligning with the results of RNA-seq (Fig. 3D). Additionally, the western blot results showed that hUCMSCs treatment reversed the low protein level of PPARγ in the liver tissues of rats in the DLC group (Fig. 3E). As a result, RNA-seq analysis of the liver tissues combined with further validation experiments indicated that PPARγ played an important role in the hUCMSCs treatment of DLC rats.

### HUCMSCs skewed the macrophage phenotype from M1-like to M2-like through the activation of PPARγ

**Fig. 4:**
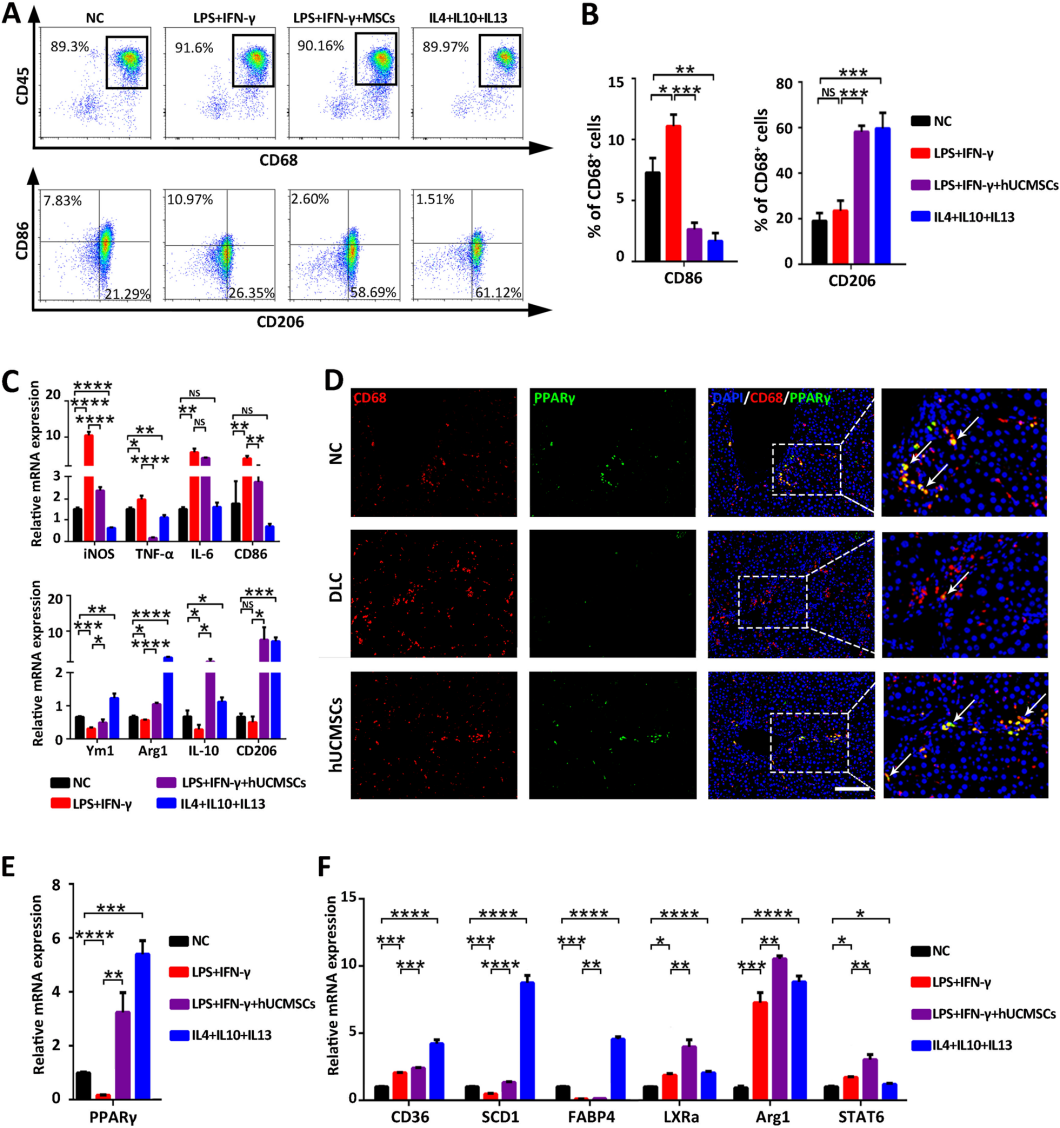
HUCMSCs promote the polarization of M2-type anti-inflammatory macrophages through the activation of PPARγ. **A**–**C**, **E**, and **F** LPS and INF-γ-induced macrophages for polarization into M1-type macrophages with or without co-culture with hUCMSCs; M2-type macrophages induced by IL4, IL10, and IL13 were used as positive controls. **A** Flow cytometry analyses of CD86 + and CD206 + macrophages in intrahepatic CD68-positive macrophages in each treatment group. **B** Statistical analysis of the percentage of CD68 + CD86 + and CD68 + CD206 + cells in each group (*n* = 3). **C** The mRNA expression of M1-related and M2-related genes in macrophages from each group was determined by RT-qPCR(*n* = 3). **D** Representative liver sections from each group stained with fluorescent CD68 (red fluorescence) and PPARγ (green fluorescence) (bar = 100 μm). **E** PPARγ mRNA expression levels in the different treatment groups (*n* = 3). **F** Downstream PPARγ mRNA expression levels in the different treatment groups (*n* = 3). Data are presented as mean ± SEM. *p < 0.05, **p < 0.01, ***p < 0.001, ****p < 0.000, and ns (no statistical significance) (all *p* values were obtained by the two-tailed Student’s test)

Based on the animal hUCMSCs treatment experiment of DLC rats that the proportion of total macrophages remained unchanged while that of the M1 type significantly decreased and that of the M2 type increased after hUCMSCs treatment. Thus, we speculated whether hUCMSCs directly affect the macrophage phenotype. To determine whether hUCMSCs would directly affect proinflammatory macrophage phenotype in vitro, primary peritoneal macrophages (marked M0) were isolated and stimulated for transformation into M1-type macrophages by LPS and IFN-γ. After that, hUCMSCs were co-cultured with M1 macrophages to observe the polarization of M1-type macrophages. Simultaneously, M2 phenotype macrophages were generated, with factors such as IL4, IL13, and IL10 as positive control. Flow cytometry revealed that the proportion of M1-type macrophages decreased from 10.97% to 2.6% after hUCMSCs treatment, whereas that of M2-type macrophages increased from 26.35% to 58.69% (Fig. 4A and B). The co-culture of hUCMSCs with macrophages decreased the expression levels of iNOS, TNF-α, CD86, and other M1-type macrophage-related genes in macrophages while increasing the expression levels of M2-type macrophage-related genes such as Ym1, Arg1, IL10 and CD206 (Fig. 4C). These results indicate that hUCMSCs can directly promote macrophage polarization from the M1-phenotype to the M2-phenotype; however, the underlying mechanism needs to be further elucidated.

Based on the results of the differential gene enrichment analysis mentioned above, we focused on the PPARγ signaling pathway, which is also enriched in immune-related differential gene clusters. PPARγ, a subtype of the peroxisome proliferator-activated receptor family, has been demonstrated to be a crucial nuclear transcription factor with anti-inflammatory function. PPARγ and its ligands have been reported to be involved in the cellular regulation of monocytes and macrophages and play an important role in the deinflammatory phase [29]. Therefore, we hypothesized that PPARγ is a potential target of hUCMSCs therapy. To validate this hypothesis, we detected the expression of PPARγ and the macrophage surface marker, CD68, in liver tissues by immunofluorescence staining of CD68 and PPARγ positive cells. Results showed that double-positive CD68 + PPARγ + cells were significantly higher in the hUCMSCs group than in the DLC group (Fig. 4D). In addition, the M1-type macrophages were treated with Rosiglitazone (Rosi), a PPARγ agonist, to verify the effect of PPARγ activation on phenotypic changes in macrophages. Flow cytometry showed that PPARγ activation could reduce the proportion of M1-type macrophages while increasing the proportion of M2-type macrophages (Additional file 1: Figure S8A). The RT-qPCR analysis confirmed these results owing to the increased mRNA expression of M2-related genes (Arg1, CD206, and CD163) and decreased expression of M1-related genes (iNOS, TNF-α, and IL-1β) after rosiglitazone treatment (Additional file 1: FigureS8B). Finally, macrophages and hUCMSCs were co-cultured to examine the direct effect of hUCMSCs on PPARγ in macrophages. The results demonstrated that the expression level of PPARγ in M1 macrophages was significantly up-regulated after co-culture with hUCMSCs (Fig. 4E). Moreover, the expression levels of downstream PPARγ genes (CD36, SCD1, FABP4, LXRa, Arg1, and STAT6) were significantly increased in the hUCMSC-treated group compared to the control group (Fig. 4F). Taken together, hUCMSCs activated PPARγ and its downstream genes in macrophages, thereby promoting the polarization of macrophages from the M1 to M2 type.

### PPARγ antagonist, GW9662, abolishes the regulation of hUCMSCs on macrophage polarization in vitro

**Fig. 5:**
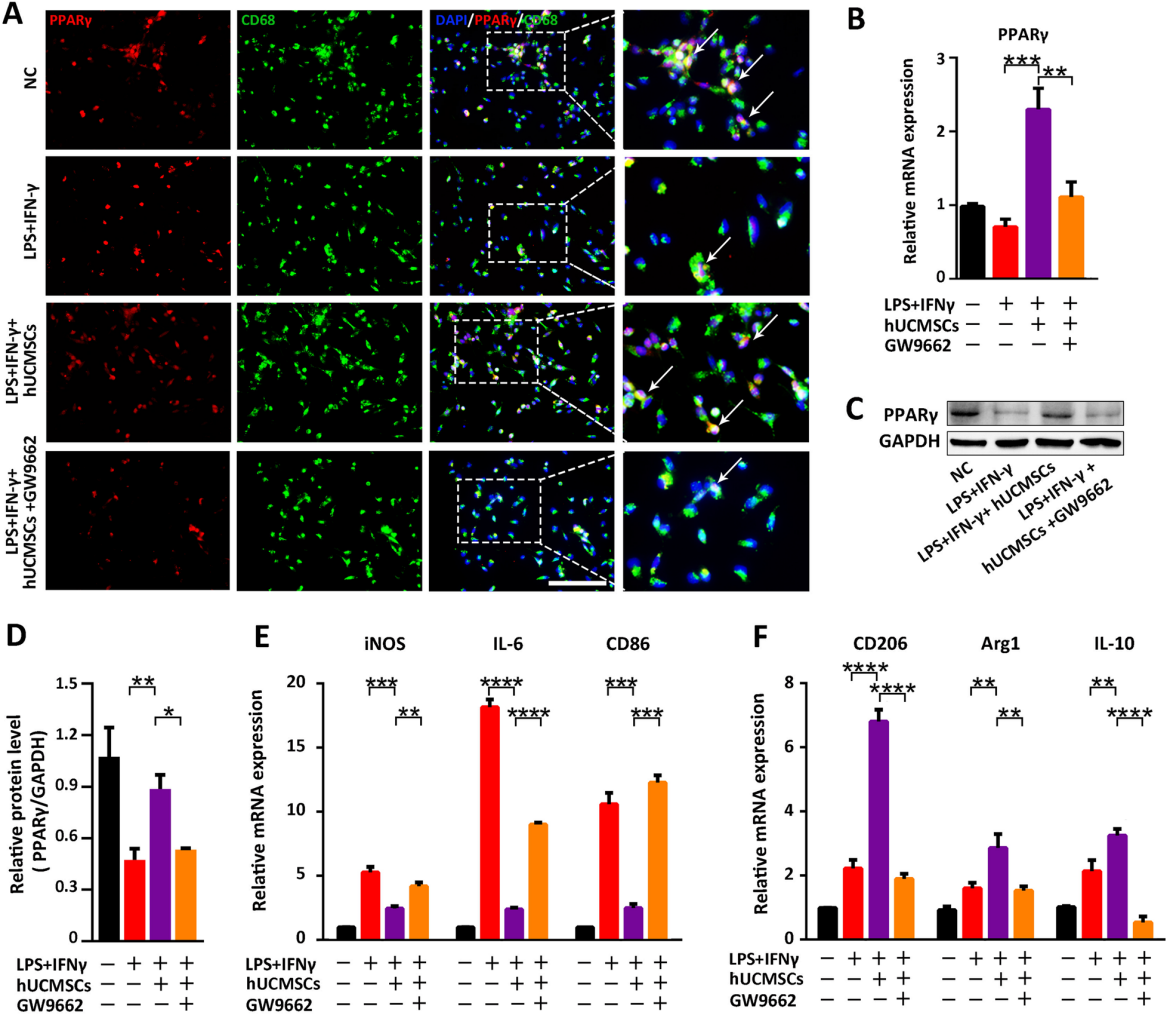
The M2-type polarization of macrophages promoted by hUCMSCSs was inhibited by the PPARγ antagonist, GW9662. **A** The fluorescence of CD68 (fluorescent green) and PPARγ (fluorescent red) was assessed to determine the level of PPARγ cells in macrophages in each group. The nucleus was stained with DAPI (fluorescent blue) (Bar = 100 μm). **B** The expression of PPARγ in macrophages was analyzed by qRT-PCR(*n* = 3). **C**–**D** PPARγ protein expression in macrophages was analyzed by Western Blot and quantitative protein analysis of PPARγ (*n* = 3) (Full-length blots and repeated experiments are presented in Additional file 3). **E** The mRNA expression levels of M1-type macrophage-related genes in macrophages (*n* = 3). **F** The mRNA expression levels of M2-type macrophage-related genes in macrophages (*n* = 3). Data are presented as mean ± SEM. *p < 0.05, **p < 0.01, ***p < 0.001, ****p < 0.0001, and ns (no statistical significance) (all *p* values were obtained by the two-tailed Student’s test)

To further investigate whether hUCMSCs affect the macrophage phenotype through the PPARγ pathway, the effect of PPARγ on the inflammatory phenotype of macrophages was determined by treating the co-cultured cells described above with the PPARγ antagonist, GW9662. The immunofluorescence assay revealed that the number of CD68 + and PPARγ + double-positive macrophages was significantly increased after co-culture with hUCMSCs, whereas the numbers of double-positive cells and fluorescence intensity were significantly decreased after the addition of the PPARγ antagonist GW9662 (Fig. 5A). RT-qPCR further proved that the up-regulated expression of macrophage PPARγ was abolished in the presence of GW9662(Fig. 5B), which aligned with the up-regulation of PPARγ protein levels in macrophages co-cultured with hUCMSCs was inhibited once treated with GW9662 (Fig. 5C and D). Additionally, the expression of M1-type and M2-type macrophage-related genes showed no significant changes in the co-culture system with the PPARγ antagonist GW9662 (Fig. 5E and F). These results confirm that hUCMSCs promoted the polarization of macrophages from pro-inflammatory M1-type to anti-inflammatory M2-type, relying on the activation of the PPARγ signaling pathway in macrophages and the anti-inflammatory treatment effects of hUCMSCs disappeared once PPARγ activation was inhibited.

### The hUCMSCs–PPARγ–macrophage axis plays a key role in DLC treatment

**Fig. 6:**
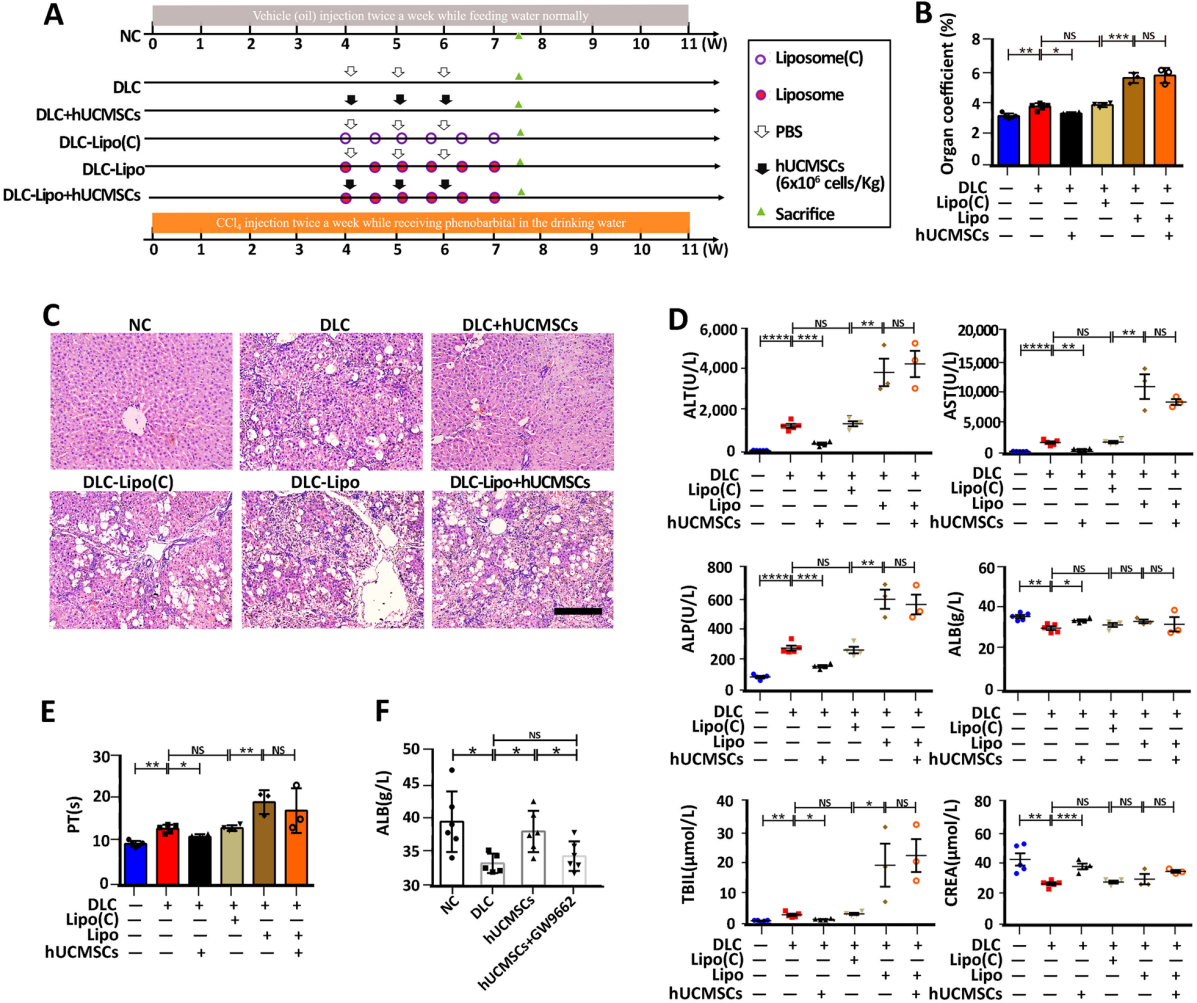
Liver macrophage exhaustion or PPARγ inhibition abolished the therapeutic effect of hUCMSCs on DLC. **A** Outline of the experimental procedure for hUCMSCs treatment in normal and macrophage-depleted DLC rats. **B** The ratio of the liver weight to the body weight for rats (NC and DLC group, *n* = 5; hUCMSC group, *n* = 4). **C** Representative sections of livers stained with H&E from six groups after different treatments (Bar = 100 μm). **D** Serum biochemical levels of ALT, AST, ALP, ALB, TBIL, and CREA (NC and DLC group, *n* = 5; DLC + hUCMSCs and DLC-Lip(C) group, *n* = 4; DLC-Lip and DLC-Lip + hUCMSCs group, *n* = 3). **E** Examination of serum PT levels (NC and DLC group, *n* = 5; DLC + hUCMSCs and DLC-Lip(C) group, *n* = 4; DLC-Lip and DLC-Lip + hUCMSCs group, *n* = 3). **F** ALB levels were measured after PPARγ inhibition (NC group, *n* = 6, DLC group, *n* = 5; hUCMSCs and hUCMSCs + GW9662 group, *n* = 6). Data are presented as mean ± SEM. *p < 0.05, **p < 0.01, ***p < 0.001, ****p < 0.0001 and ns (no statistical significance) (all *p* values were obtained by Two-tailed Student’s Test)

Although several studies have reported the therapeutic effects of hUCMSCs on cirrhosis involving macrophages, the mechanisms underlying the progression of cirrhosis are not completely understood. In this study, we confirmed the macrophage phenotype switches under hUCMSCs treatment in vivo and in vitro; however, whether this effect plays a crucial role in DLC disease progression and hUCMSCs treatment needs further exploration. Therefore, macrophages were depleted using clodronate liposomes during hUCMSCs treatment to investigate whether the repair effect of DLC treatment on the liver was partially affected by macrophages. The acquisition and treatment regimen of macrophage-depleted DLC rats are shown in Fig. 6A; macrophage depletion was the only difference between the DLC + hUCMSCs and DLC-Liposome + hUCMSCs groups. We observed that macrophage depletion using liposomes significantly decreased the proportion of macrophages in the liver and blood of DLC rats, suggesting the successful establishment of the DLC rats with hepatic macrophage depletion (Additional file 1: Figure S9A and B). Further investigation revealed that hUCMSCs treatment could significantly promote the body weight increase of DLC rats, but could not improve the rapidly decreasing body weight of macrophage-depleted DLC rats (Additional file 1: Figure S9C). The livers of rats in the macrophage-depleted group were swollen, rough in texture, and stiff, with small nodules. Furthermore, there was no significant difference in the appearance of the liver after hUCMSCs treatment and the liver organ coefficient also showed no differences (Additional file 1: Figure S9D and Fig. 6B). What’s more, HE staining results showed that hUCMSCs treatment could not restore the damaged liver structure, improve the fatty degeneration of the liver, and reduce the inflammatory infiltration in the liver, and hepatonecrosis in the DLC-Lipsome group (Fig. 6C). SR staining further confirmed hUCMSCs treatment could not reduce collagen deposition in the DLC-Lipsome group (Additional file 1: Figure S9E). Evidently, the administration of hUCMSCs in the macrophage-depleted DLC-Lipsome group failed to reduce the serum biochemical indexes, including ALT, AST, ALP, PT, and TBIL; increase the liver function indexes, such as ALB and CREA levels; and improve PT coagulation function (Fig. 6D and E). All these results indicate that the depletion of intrahepatic macrophages aggravates disease progression in DLC rats, and hUCMSCs treatment cannot improve the disease characteristics in macrophage-depleted DLC rats.

As mentioned above, activation of the PPARγ pathway has been proved to promote the polarization of M2 macrophages in vitro. However, whether the activation of PPARγ plays an important role in DLC rats with hUCMSCs treatment needs to be further elucidated. Consequently, the PPARγ antagonist, GW9662, was employed during hUCMSCs treatment to induce the systemic inhibition of PPARγ in DLC rats. HE staining revealed that the damaged liver structure and hepatonecrosis did not significantly improve, with worse inflammatory infiltration in DLC rats treated with hUCMSCs and GW9662 (Additional file 1: Figure S10A). Moreover, Sirian red staining revealed no decreased collagen fibers in DLC rats treated with hUCMSCs and GW9662 compared with DLC rats only treated with hUCMSCs (Additional file 1: Figure S10B). In addition, the level of serum ALB, which can reflect the synthetic protein function of the liver, was significantly up-regulated to 38.2 ± 4.2 g/L in the hUCMSCs group, while a difference was not found between the hUCMSCs group treated with GW9662 (34.8 ± 3 g/L) and the DLC group(33.6 ± 1.6 g/L) (Fig. 6F). Based on these results, the inhibition of PPARγ attenuated the beneficial effect of hUCMSCs treatment in DLC rats, suggesting that the activation of the PPARγ pathway plays an indispensable role in hUCMSCs treatment in DLC rats.

**Fig. 7:**
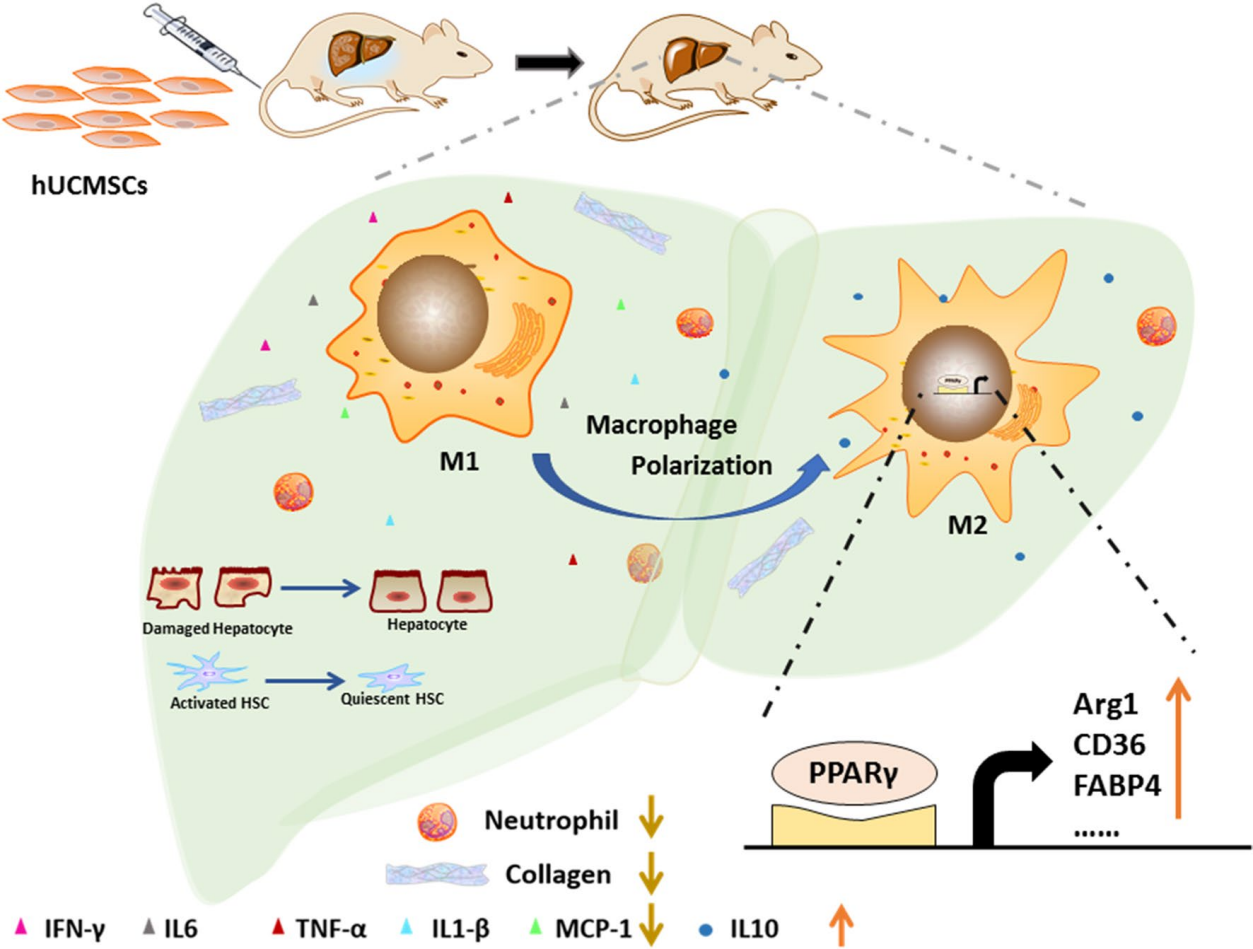
Hepatoprotective and immunoregulative effects of hUCMSCs in DLC rats. Schematic diagram showing the potential molecular mechanisms through which hUCMSCs alleviate hepatocellular damage by promoting the polarization of M2 type macrophages through PPARγ activation and the inhibition of inflammatory response in DLC rats

In summary, a schematic diagram of the mechanisms of hUCMSCs treatment in DLC rats is shown in Fig. 7. Briefly, hUCMSCs treatment can reconstruct liver structure, reduce ascites, hepatocyte necrosis, neutrophil infiltration, and collagen deposition, inhibit the activation of hepatic stellate cells, and decrease the expression level of inflammatory factors in DLC rats. Mechanistically, hUCMSCs treatment induces the polarization of proinflammatory macrophages into repair macrophages by activating the nuclear transcription factor, PPARγ, thereby eliminating the inflammatory response and promoting tissue repair.

## Discussion

Owing to the growing burden of liver diseases worldwide, the prevention and treatment of patients with decompensated liver cirrhosis will become a substantial healthcare challenge. Among the different types of therapies, hUCMSCs transplantation has emerged as the best alternative to liver transplantation for treating DLC. However, the optimal treatment regimens and molecular mechanisms of hUCMSCs on DLC remain unclear [26, 30, 31]. The findings of this study not only confirmed the excellent effects found in previous studies but also revealed that the optimal hUCMSCs infusion regimen is weekly infusion for three consecutive weeks at the early stage of DLC rats, and the mechanism of hUCMSCs treatment is the polarization of pro-inflammatory M1-type macrophages to anti-inflammatory M2-type through PPARγ activation.

In this study, our results demonstrated that hUCMSCs infusion at week 8 of modeling, corresponding to the end-stage of DLC, had a specific therapeutic effect on DLC. Notably, repeat hUCMSCs infusion in the T-B group (once per week for three consecutive weeks) at the early stage of DLC (week 5 of modeling) can significantly improve the efficacy of hUCMSCs treatment in DLC, and mainly prevent ascites production and death of DLC rats. The multifaceted efficacies of hUCMSCs are as follows 1. Reduce the necrosis of liver cells and the release of cytoplasmic enzymes ALT, AST, and ALP; 2. Improve liver function, increase albumin, decrease total bilirubin and prothrombin time; 3. Improve the liver microenvironment, reduce the level of inflammatory factors, and increase the expression of the anti-inflammatory factor, IL10; 4. Reduce fiber hyperplasia and restore the structural disorder caused by fiber septum; Based on our treatment regimen results, the infusion starting time and infusion times should be considered in clinical hUCMSCs therapy on DLC. Of note, the infusion interval might be insignificant.

A clinical observational study demonstrated that extending the treatment course (more than four times) may be an option to improve the efficacy of hUCMSCs, aligning with our results that three injections of hUCMSCs were superior to one injection at the early stage of DLC [32]. Surprisingly, three injections of hUCMSCs were less efficacious than one infusion at the end-stage of DLC, indicating that the disease stage must be considered in the formulation of the frequency schedule for hUCMSCs treatment. Most clinical trials focus on the dosage and duration of MSCs treatment while ignoring the initial infusion time points corresponding to clinical characteristics [6, 33–35]. Our results strongly support that hUCMSCs infusion combined with the optimized initial infusion time and frequency could be a very promising treatment approach for DLC. Importantly, hUCMSC-based therapy should not only be regarded as the last option for treating advanced diseases but should also be combined with other conventional treatments as early as possible to improve its therapeutic effect in patients with DLC. Although a certain gap exists between the treatment benefits in animal models and clinical applications, our findings enrich the preclinical study of MSCs treatment for DLC, providing an important reference for future clinical treatment.

To evaluate the effects of hUCMSCs-infusion on DLC, a rat model that largely conforms to the typical clinical features of DLC was established in this study. To date, no animal model has completely demonstrated the disease progression of decompensated cirrhosis through continuous weekly monitoring of the DLC model. Although the combination of phenobarbital and CCl_4_ has become the standard method to construct decompensated cirrhosis animal models, as reported previously, only a few indexes are used to evaluate after the modeling completion [36–39]. Consequently, comprehensive evaluation and dynamic monitoring of disease occurrence and development process still need to be improved. More importantly, the lack of preclinical animal models to closely simulate the clinical and pathologic features of human DLC has limited the execution of studies on the therapeutic mechanism of MSCs and the improvement of clinical treatment regimens. In contrast to previous reports that defined experimental DLC using a single or few indicators [40–42], we performed real-time dynamic detection of the liver structure, degree of liver fibrosis, liver function, and the amount of ascites in rats for 11 weeks, and fully understood the stage of disease development and the corresponding disease characteristics of DLC in DLC rats. Based on our results, 5–7 weeks of rat modeling could correspond to the early formative stage of DLC in patients, and 8–11 weeks of rat modeling could be considered to correspond to the end stage of DLC in patients, thereby laying a solid foundation for further studies on the treatment and mechanism of decompensated cirrhosis. In addition, to observe the homing effect of hUCMSCs in DLC model rats, intravenous injection of hUCMSCs at week 5 of modeling revealed that hUCMSCs mainly homed in the damaged liver, as shown in Additional file 1: Figure S2, which is consistent with other studies on MSCs homing [43–45].

MSCs exert immunomodulatory and immunosuppressive effects in vivo and in vitro [3, 46]. In several mouse models of acute liver failure, MSCs significantly reduce inflammatory cell infiltration, reduce apoptosis, and increase hepatocyte proliferation, thus promoting liver regeneration and improving survival [47–49]. MSCs have been reported to modulate adaptive immune responses, induce DC to up-regulate the anti-inflammatory cytokine, IL-10, and reduce the secretion of the pro-inflammatory cytokines, TNF-α and IL-12 [50]. In this study, hUCMSCs treatment changed the immune microenvironment of the liver based on a significant decrease in neutrophils compared with that in the DLC model group, a decrease in the proportion of M1 macrophages, and an increase in the proportion of M2 macrophages. Accordingly, we will investigate the dynamic effects on liver macrophages after hUCMSCs infusion to further clarify the therapeutic mechanism of hUCMSCs on macrophages, as the activity of immune cells was found to vary at different stages of the disease.

Hepatic macrophages, including resident Kupffer cells (KCs) and recruited monocyte-derived macrophages, are highly plastic and can adjust their phenotypes according to signals from the microenvironment [51]. MSCs treatment restores liver macrophage homeostasis to ameliorate the progression of liver diseases including acute liver injury, liver fibrosis, and cirrhosis [52–54]. Consistently, different hUCMSCs infusion regimens were found to exhibit differential effects on macrophage homeostasis regulation ability, among which the T-B group could significantly down-regulate the M1/M2 macrophage ratio. Apparently, our findings suggest that macrophage polarization plays a central role in hUCMSCs therapy for decompensated cirrhosis and the therapeutic effect of hUCMSCs affected by infusion time point was highly correlated with M1/M2 polarization.

According to previous studies, PPARγ, which is essential for macrophage polarization, is involved in regulating of M1-type macrophage polarization to M2-type in inflammation and injury-related diseases, such as Parkinson’s disease, sepsis, and atherosclerosis [55–57]. Besides, the activation of PPARγ promotes the polarization of M2-type macrophages to prevent the development of non-alcoholic fatty liver disease and liver injury [27, 58, 59]. Recent studies show that the expression of PPARγ in macrophages was up-regulated in the cytoplasm initially, and then, nuclear translocation occurred, and finally, activating the downstream signaling pathway [60, 61]. In this study, hUCMSCs were found to induce the polarization of M1-type macrophages into M2-type macrophages by activating PPARγ in vivo and in vitro. However, whether the activation of PPARγ pathway attributed to nuclear translocation of up-regulated PPARγ after hUCMSC treatment in DLC rats remains to be further elucidated. Previous studies on PPARγ-deficient macrophages revealed their resistance to M2 polarization [62]. Consistently, our findings revealed that hUCMSCs infusion had no therapeutic effect in DLC rats after intrahepatic macrophages depletion with clodronate liposomes, which was validated by the significantly reduced therapeutic effect of hUCMSCs after injection of GW9662 (PPARγ antagonist) during the DLC treatment period, proving that the existence and activation of macrophages PPARγ are indispensable for DLC therapy. This study had some limitations as we only verified that the effect of hUCMSCs on macrophage polarization disappeared when PPARγ antagonists were used in vitro; however, further verification was not carried out in macrophage-specific PPARγ knockout rats.

## Conclusions

In conclusion, we examined the DLC rat model for 11 consecutive weeks and assessed the typical clinical features corresponding to each disease stage; according to that, we formulated the different hUCMSCs infusion regimens and found that the optimal hUCMSCs infusion regimen was once per week for three consecutive weeks at the early stage of DLC. This regimen can effectively inhibit the occurrence of ascites and significantly improve liver structure and function in DLC rats. The therapeutic mechanism of hUCMSCs is mainly attributed to the polarization of M1-type macrophages to M2-type macrophages through the activation of the PPARγ signaling pathway in DLC rat liver macrophages, thereby inhibiting inflammation and promoting the repair of damaged liver tissue. This study laid a solid experimental foundation for elucidating the functions and mechanisms of hUCMSCs treatment in DLC. At the same time, our studies on hUCMSCs-based therapy with optimal regimens reveal that this therapy might serve as an effective alternative to fulfill the needs for the treatment of DLC, ultimately providing a new approach for promoting and advancing the clinical application of cell-based therapy.

## Supplementary Information

**Additional file 1:** Supplementary Tabels and Figures.

**Additional file 2:** Original blot images of Figure 3E.

**Additional file 3:** Original blot images of Figure 5C.

## Data Availability

All RNA-seq raw data analyzed in this study are deposited in the NCBI SRA database (Accession No. SRP287012) (https://www.ncbi.nlm.nih.gov/sra/?term=SRP287012).

## Abbreviations

DLC: Decompensated liver cirrhosis
MSCs: Mesenchymal stem cells
hUCMSCs: Human umbilical cord mesenchymal stem cells
ALT: Alanine aminotransferase
AST: Aspartate aminotransferase
ALP: Alkaline phosphatase
ALB: Albumin
α-SMA: Alpha smooth muscle Actin
CCl_4_: Carbon tetrachloride
Col1a1: Collagen type I alpha 1
CREA: Creatinine
DLC: Decompensated liver cirrhosis
GGT: Gamma-glutamyl transpeptidase
GO: Gene ontology
HRP: Horseradish peroxidase
H&E: Hematoxylin and eosin staining
IL-6: Interleukin-6
IL-10: Interleukin-10
IFN-γ: Interferon-γ
KEGG: Kyoto Encyclopedia of Genes and Genomes
PB: Phenobarbital
PPARγ: Peroxisome proliferator-activated receptor γ
PT: Prothrombin time
TBIL: Total bilirubin
TGF-β1: Transforming growth factor β1
TNF-α: Tumor necrosis factor-α
WB: Western blot

## References

[1] Shiels M, Chernyavskiy P, Anderson W, Best A, Haozous E, Hartge P, Rosenberg P, Thomas D, Freedman N, Berrington de Gonzalez A (2017). Trends in premature mortality in the USA by sex, race, and ethnicity from 1999 to 2014: an analysis of death certificate data. Lancet (London, England).

[2] D'Amico G, Pasta L, Morabito A, D'Amico M, Caltagirone M, Malizia G, Tinè F, Giannuoli G, Traina M, Vizzini G (2014). Competing risks and prognostic stages of cirrhosis: a 25-year inception cohort study of 494 patients. Aliment Pharmacol Ther.

[3] Uccelli A, Moretta L, Pistoia V (2008). Mesenchymal stem cells in health and disease. Nat Rev Immunol.

[4] Sattwika P, Indrarti F, Bayupurnama P (2021). Clinical application of stem cell therapy for liver cirrhosis: progress, pitfalls, and prospects. Acta Med Indones.

[5] Alfaifi M, Eom YW, Newsome PN, Baik SK (2018). Mesenchymal stromal cell therapy for liver diseases. J Hepatol.

[6] Liu Y, Dong Y, Wu X, Xu X, Niu J (2022). The assessment of mesenchymal stem cells therapy in acute on chronic liver failure and chronic liver disease: a systematic review and meta-analysis of randomized controlled clinical trials. Stem Cell Res Ther.

[7] Sharma M, Pondugala P, Jaggaihgari S, Mitnala S, Krishna V, Jaishetwar G, Naik P, Kumar P, Kulkarni A, Gupta R (2022). Safety assessment of autologous stem cell combination therapy in patients with decompensated liver cirrhosis: a pilot study. J Clin Exp Hepatol.

[8] Suk K, Yoon J, Kim M, Kim C, Kim J, Park H, Hwang S, Kim D, Lee B, Lee S (2016). Transplantation with autologous bone marrow-derived mesenchymal stem cells for alcoholic cirrhosis: phase 2 trial. Hepatology (Baltimore, MD).

[9] Braun F, Rinschen M, Buchner D, Bohl K, Plagmann I, Bachurski D, Richard Späth M, Antczak P, Göbel H, Klein C (2020). The proteomic landscape of small urinary extracellular vesicles during kidney transplantation. J Extracell Vesicles.

[10] Salama H, Zekri A, Medhat E, Al Alim S, Ahmed O, Bahnassy A, Lotfy M, Ahmed R, Musa S (2014). Peripheral vein infusion of autologous mesenchymal stem cells in Egyptian HCV-positive patients with end-stage liver disease. Stem Cell Res Ther.

[11] Shi M, Li Y, Xu R, Meng F, Yu S, Fu J, Hu J, Li J, Wang L, Jin L (2021). Mesenchymal stem cell therapy in decompensated liver cirrhosis: a long-term follow-up analysis of the randomized controlled clinical trial. Hep Intl.

[12] Rajaram R, Subramani B, Abdullah B, Mahadeva S (2017). Mesenchymal stem cell therapy for advanced liver cirrhosis: a case report. JGH Open Open Access J Gastroenterol Hepatol.

[13] Mohamadnejad M, Alimoghaddam K, Bagheri M, Ashrafi M, Abdollahzadeh L, Akhlaghpoor S, Bashtar M, Ghavamzadeh A, Malekzadeh R (2013). Randomized placebo-controlled trial of mesenchymal stem cell transplantation in decompensated cirrhosis. Liver Int Off J Int Assoc Study Liver.

[14] Najar M, Raicevic G, Boufker H, Fayyad Kazan H, De Bruyn C, Meuleman N, Bron D, Toungouz M, Lagneaux L (2010). Mesenchymal stromal cells use PGE2 to modulate activation and proliferation of lymphocyte subsets: combined comparison of adipose tissue, Wharton's Jelly and bone marrow sources. Cell Immunol.

[15] Liu R, Zhang Z, Lu Z, Borlongan C, Pan J, Chen J, Qian L, Liu Z, Zhu L, Zhang J (2013). Human umbilical cord stem cells ameliorate experimental autoimmune encephalomyelitis by regulating immunoinflammation and remyelination. Stem Cells Dev.

[16] Baksh D, Yao R, Tuan R (2007). Comparison of proliferative and multilineage differentiation potential of human mesenchymal stem cells derived from umbilical cord and bone marrow. Stem cells (Dayton, Ohio).

[17] Deng Y, Zhang Y, Ye L, Zhang T, Cheng J, Chen G, Zhang Q, Yang Y (2016). Umbilical cord-derived mesenchymal stem cells instruct monocytes towards an IL10-producing phenotype by secreting IL6 and HGF. Sci Rep.

[18] Wang P, Cui Y, Wang J, Liu D, Tian Y, Liu K, Wang X, Liu L, He Y, Pei Y (2022). Mesenchymal stem cells protect against acetaminophen hepatotoxicity by secreting regenerative cytokine hepatocyte growth factor. Stem Cell Res Ther.

[19] Meier R, Mahou R, Morel P, Meyer J, Montanari E, Muller Y, Christofilopoulos P, Wandrey C, Gonelle-Gispert C, Bühler L (2015). Microencapsulated human mesenchymal stem cells decrease liver fibrosis in mice. J Hepatol.

[20] An S, Jang Y, Lim H, Han J, Lee J, Lee G, Park J, Park S, Kim J, Do B (2017). Milk fat globule-EGF factor 8, secreted by mesenchymal stem cells, protects against liver fibrosis in mice. Gastroenterology.

[21] Zhang Z, Lin H, Shi M, Xu R, Fu J, Lv J, Chen L, Lv S, Li Y, Yu S (2012). Human umbilical cord mesenchymal stem cells improve liver function and ascites in decompensated liver cirrhosis patients. J Gastroenterol Hepatol.

[22] Das D, Paul A, Lahiri A, Adak M, Maity S, Sarkar A, Paul S, Chakrabarti P (2021). Proteasome dysfunction under compromised redox metabolism dictates liver injury in NASH through ASK1/PPARγ binodal complementary modules. Redox Biol.

[23] Skat-Rørdam J, Højland Ipsen D, Lykkesfeldt J, Tveden-Nyborg P (2019). A role of peroxisome proliferator-activated receptor γ in non-alcoholic fatty liver disease. Basic Clin Pharmacol Toxicol.

[24] Polyzos S, Bugianesi E, Kountouras J, Mantzoros C (2017). Nonalcoholic fatty liver disease: updates on associations with the metabolic syndrome and lipid profile and effects of treatment with PPAR-γ agonists. Metabol Clin Exp.

[25] Mathew SA, Naik C, Cahill PA, Bhonde RR (2020). Placental mesenchymal stromal cells as an alternative tool for therapeutic angiogenesis. Cell Mol Life Sci.

[26] Heo JS, Choi Y, Kim HS, Kim HO (2016). Comparison of molecular profiles of human mesenchymal stem cells derived from bone marrow, umbilical cord blood, placenta and adipose tissue. Int J Mol Med.

[27] Gong W, Zhu H, Lu L, Hou Y, Dou H (2019). A benzenediamine analog FC-99 drives M2 macrophage polarization and alleviates lipopolysaccharide- (LPS-) induced liver injury. Mediat Inflamm.

[28] McMullen M, Pritchard M, Nagy L (2008). Isolation of Kupffer cells from rats fed chronic ethanol. Methods Mol Biol (Clifton N.J.).

[29] Croasdell A, Duffney PF, Kim N, Lacy SH, Sime PJ, Phipps RP (2015). PPARγ and the innate immune system mediate the resolution of inflammation. PPAR Res.

[30] Gazdic M, Arsenijevic A, Markovic B, Volarevic A, Dimova I, Djonov V, Arsenijevic N, Stojkovic M, Volarevic V (2017). Mesenchymal stem cell-dependent modulation of liver diseases. Int J Biol Sci.

[31] Lee C, Chen Y, Wu H, Lee O (2018). Historical perspectives and advances in mesenchymal stem cell research for the treatment of liver diseases. Gastroenterology.

[32] Jia Y, Shu X, Yang X, Sun H, Cao H, Cao H, Zhang K, Xu Q, Li G, Yang Y (2020). Enhanced therapeutic effects of umbilical cord mesenchymal stem cells after prolonged treatment for HBV-related liver failure and liver cirrhosis. Stem Cell Res Ther.

[33] Schacher F, Martins Pezzi da Silva A, Silla L, Álvares-da-Silva M (2021). Bone marrow mesenchymal stem cells in acute-on-chronic liver failure grades 2 and 3: a phase I-II randomized clinical trial. Can J Gastroenterol Hepatol.

[34] Xu W, He H, Pan S, Chen Y, Zhang M, Zhu S, Gao Z, Peng L, Li J (2019). Combination treatments of plasma exchange and umbilical cord-derived mesenchymal stem cell transplantation for patients with hepatitis b virus-related acute-on-chronic liver failure: a clinical trial in china. Stem Cells Int.

[35] Kharaziha P, Hellström PM, Noorinayer B, Farzaneh F, Aghajani K, Jafari F, Telkabadi M, Atashi A, Honardoost M, Zali MR (2009). Improvement of liver function in liver cirrhosis patients after autologous mesenchymal stem cell injection: a phase I-II clinical trial. Eur J Gastroenterol Hepatol.

[36] Nishikawa T, Bell A, Brooks J, Setoyama K, Melis M, Han B, Fukumitsu K, Handa K, Tian J, Kaestner K (2015). Resetting the transcription factor network reverses terminal chronic hepatic failure. J Clin Investig.

[37] O'Brien A, Fullerton J, Massey K, Auld G, Sewell G, James S, Newson J, Karra E, Winstanley A, Alazawi W (2014). Immunosuppression in acutely decompensated cirrhosis is mediated by prostaglandin E2. Nat Med.

[38] Kobayashi N, Ito M, Nakamura J, Cai J, Gao C, Hammel J, Fox I (2000). Hepatocyte transplantation in rats with decompensated cirrhosis. Hepatology (Baltimore, MD).

[39] Kim S, Schou U, Peters C, de Seigneux S, Kwon T, Knepper M, Jonassen T, Frøkiaer J, Nielsen S (2005). Increased apical targeting of renal epithelial sodium channel subunits and decreased expression of type 2 11beta-hydroxysteroid dehydrogenase in rats with CCl4-induced decompensated liver cirrhosis. J Am Soc Nephrol.

[40] Lin B, Chen J, Qiu W, Wang K, Xie D, Chen X, Liu Q, Peng L, Li J, Mei Y (2017). Allogeneic bone marrow-derived mesenchymal stromal cells for hepatitis B virus-related acute-on-chronic liver failure: a randomized controlled trial. Hepatology (Baltimore, MD).

[41] Suk KT, Yoon JH, Kim MY, Kim CW, Kim JK, Park H, Hwang SG, Kim DJ, Lee BS, Lee SH (2016). Transplantation with autologous bone marrow-derived mesenchymal stem cells for alcoholic cirrhosis: phase 2 trial. Hepatology.

[42] Shi M, Zhang Z, Xu R, Lin H, Fu J, Zou Z, Zhang A, Shi J, Chen L, Lv S (2012). Human mesenchymal stem cell transfusion is safe and improves liver function in acute-on-chronic liver failure patients. Stem Cells Transl Med.

[43] Leibacher J, Henschler R (2016). Biodistribution, migration and homing of systemically applied mesenchymal stem/stromal cells. Stem Cell Res Ther.

[44] Nitzsche F, Müller C, Lukomska B, Jolkkonen J, Deten A, Boltze J (2017). Concise review: MSC adhesion cascade-insights into homing and transendothelial migration. Stem Cells.

[45] Yuan M, Hu X, Yao L, Jiang Y, Li L (2022). Mesenchymal stem cell homing to improve therapeutic efficacy in liver disease. Stem Cell Res Ther.

[46] Mezey É (2022). Human mesenchymal stem/stromal cells in immune regulation and therapy. Stem Cells Transl Med.

[47] van Poll D, Parekkadan B, Cho CH, Berthiaume F, Nahmias Y, Tilles AW, Yarmush ML (2008). Mesenchymal stem cell-derived molecules directly modulate hepatocellular death and regeneration in vitro and in vivo. Hepatology.

[48] Huang B, Cheng X, Wang H, Huang W, la Ga Hu Z, Wang D, Zhang K, Zhang H, Xue Z, Da Y (2016). Mesenchymal stem cells and their secreted molecules predominantly ameliorate fulminant hepatic failure and chronic liver fibrosis in mice respectively. J Transl Med.

[49] Shokravi S, Borisov V, Zaman BA, Niazvand F, Hazrati R, Khah MM, Thangavelu L, Marzban S, Sohrabi A, Zamani A (2022). Mesenchymal stromal cells (MSCs) and their exosome in acute liver failure (ALF): a comprehensive review. Stem Cell Res Ther.

[50] Aggarwal S, Pittenger MF (2005). Human mesenchymal stem cells modulate allogeneic immune cell responses. Blood.

[51] Krenkel O, Tacke F (2017). Liver macrophages in tissue homeostasis and disease. Nat Rev Immunol.

[52] Zhou J, Feng X, Zhu J, Feng B, Yao Q, Pan Q, Yu J, Yang J, Li L, Cao H (2022). Mesenchymal stem cell treatment restores liver macrophages homeostasis to alleviate mouse acute liver injury revealed by single-cell analysis. Pharmacol Res.

[53] Li Y, Shen S, Shao T, Jin M, Fan D, Lin A, Xiang L, Shao J (2021). Mesenchymal stem cells attenuate liver fibrosis by targeting Ly6C macrophages through activating the cytokine-paracrine and apoptotic pathways. Cell death discovery.

[54] Nojiri S, Tsuchiya A, Natsui K, Takeuchi S, Watanabe T, Kojima Y, Watanabe Y, Kamimura H, Ogawa M, Motegi S (2021). Synthesized HMGB1 peptide attenuates liver inflammation and suppresses fibrosis in mice. Inflamm Regen.

[55] Ji J, Xue TF, Guo XD, Yang J, Guo RB, Wang J, Huang JY, Zhao XJ, Sun XL (2018). Antagonizing peroxisome proliferator-activated receptor γ facilitates M1-to-M2 shift of microglia by enhancing autophagy via the LKB1-AMPK signaling pathway. Aging Cell.

[56] Huang T, Wu H, Chen S, Wang Y, Wu C (2020). Thrombomodulin facilitates peripheral nerve regeneration through regulating M1/M2 switching. J Neuroinflammation.

[57] Van der Vorst, E., Biessen, E. Unwrapped and uNCORked: PPAR-γ repression in atherosclerosis. European Heart Journal 2019. 10.1093/eurheartj/ehz770 31754688

[58] Luo W, Xu Q, Wang Q, Wu H, Hua J (2017). Effect of modulation of PPAR-γ activity on Kupffer cells M1/M2 polarization in the development of non-alcoholic fatty liver disease. Sci Rep.

[59] Zhong X, Liu H (2018). Honokiol attenuates diet-induced non-alcoholic steatohepatitis by regulating macrophage polarization through activating peroxisome proliferator-activated receptor γ. J Gastroenterol Hepatol.

[60] Zhao M, Bian YY, Yang LL, Chen YQ, Wang YJ, Ma YT, Pei YQ, Li WL, Zeng L (2019). HuoXueTongFu formula alleviates intraperitoneal adhesion by regulating macrophage polarization and the SOCS/JAK2/STAT/PPAR-γ signalling pathway. Mediators Inflamm.

[61] Luo J, Wang J, Zhang J, Sang A, Ye X, Cheng Z, Li X (2022). Nrf2 deficiency exacerbated CLP-induced pulmonary injury and inflammation through autophagy- and NF-κB/PPARγ-mediated macrophage polarization. Cells.

[62] Odegaard JI, Ricardo-Gonzalez RR, Goforth MH, Morel CR, Subramanian V, Mukundan L, Red Eagle A, Vats D, Brombacher F, Ferrante AW (2007). Macrophage-specific PPARgamma controls alternative activation and improves insulin resistance. Nature.

