# Supplementary material for: PPARγ-dependent hepatic macrophage switching acts as a central hub for hUCMSC-mediated alleviation of decompensated liver cirrhosis in rats
Source: Stem Cell Res Ther. 2023 Jul 27;14:184. doi: 10.1186/s13287-023-03416-2 (PMC10375757; doi:10.1186/s13287-023-03416-2)
Supplement: Supplementary file 1 — Additional file 1: Supplementary Tabels and Figures. [file 13287_2023_3416_MOESM1_ESM.pdf]

Supplementary Tables:

TableS1: Primers sequences used in this study:

Primers	Sequence (5' to 3')
β-actin-Forward	CTAAGGCCAACCGTGAAAAGATG
β-actin-Reverse	TACGACCAGAGGCATACAGG
IL-1β-Forward	AGGCTGACAGACCCCAAAAG
IL-1β-Reverse	CTCCACGGGCAAGACATAGG
iNOS/NOS2-Forward	GGAGAAAACCCCAGGTGCTAT
iNOS/NOS2-Reverse	TGAGGAACTGGGGGAAACCA
Ccl2/MCP-1-Forward	CTGTGCTGACCCCAATAAGGA
Ccl2/MCP-1-Reverse	ACAGAAGTGCTTGAGGTGGT
Cxcl2/MIP-2-Forward	ATGCTGTACTGGTCCTGCTC
Cxcl2/MIP-2-Reverse	GTAGGGTCGTCAGGCATTGA
IL-6-Forward	TGCCTTCTTGGGACTGATGT
IL-6-Reverse	TGGTCTGTTGTGGGTGGTATC
HNF1a-Forward	GACGTCTCCAGGTCTCAACC
HNF1a-Reverse	CACCCGTGTTAGTGAACGTG
CD163-Forward	CCTGGTTTGTGGAGCCATTC
CD163-Reverse	GGTTTCTTAAATGCCAACCCGA
Arg1-Forward	TTTGGGTGGATGCTCACACT
Arg1-Reverse	ACACGATGTCCTTGGCAGAT
IL-10-Forward	GGGAGAGAAGCTGAAGACCC
IL-10-Reverse	TTGAGTGTCACGTAGGCTTCT
Stat3-Forward	AAGCTGACCCAGGTAGTGCT
Stat3-Reverse	TCCATGTCAAACGTGAGCGA
C/EBPβ-Forward	GAGCGACGAGTACAAGATGC
C/EBPβ-Reverse	CAGCTGCTTGAACAAGTTCCG
C/EBPα-Forward	GGGAACGCAACAACATCGC
C/EBPα-Reverse	ACTGGTCAACTCCAACACCT
TNF-Forward	GATCGGTCCCAACAAGGAGG
TNF-Reverse	TTTGCTACGACGTGGGCTAC
Ppara-Forward	AATGCAATCCGTTTTGGAAG
Ppara-Reverse	GCCAGAGATTTGAGGTCTGC
CD86-Forward	TGCTGTCTCTCTCTGCTGACG
CD86-Reverse	GCTCGGACTCACGAGTCTTTC
CD206-Forward	ACACACTGGAACGCTGACAT
CD206-Reverse	TCCCATAAACCACCTGCCAC
Ym1-Forward	GATGGGCATGAGGGTAGCACT
Ym1-Reverse	CCATTGCCTTCCCGGTACT
CD36-Forward	TTTGTTCTTCCAGCCAACGC
CD36-Reverse	TGTCCAGCACACCATACGAC
SCD1-Forward	TCTCGGGAGAACATCCTGGT
SCD1-Reverse	AAGTTGATGTGCCAGCGGTA
FABP4-Forward	AGAAGTGGGAGTTGGCTTCG
FABP4-Reverse	ACTCTCTGACCGGATGACGA
LXRα-Forward	GGGAACGAGCTATGCAGTGT
LXRα-Reverse	GAAGAATCCCTTGCAGCCCT

STAT6-Forward	AGGATGAGGCTTTTCGGAGC
STAT6-Reverse	ATCTGGGGCTCTGGAGTAGG
CD68-Forward	AGCAGCACAGTGGACATTCT
CD68-Reverse	CAAGAGAAGCATGGCCCGAA

TableS2: Antibodies used in this study

Antibodies	Source	Identifier
Antibodies for flow cytometry		
Anti-human CD14	Biolegend	Cat#325605
Anti-human CD34	Biolegend	Cat#343504
Anti-human CD45	Biolegend	Cat#304008
Anti-human CD11b	Biolegend	Cat#301306
Anti-human HLA-DR	Biolegend	Cat#307606
Anti-human CD29	Biolegend	Cat#303004
Anti-human CD44	Biolegend	Cat#338808
Anti-human CD73	Biolegend	Cat#344004
Anti-human CD90	Biolegend	Cat#328110
Anti-human CD105	Biolegend	Cat#323206
Anti-Rat CD45	Biolegend	Cat#202221
Anti-Rat CD3	Biolegend	Cat#201413
Anti-Rat CD4	Biolegend	Cat#203305
Anti-Rat CD8	Biolegend	Cat#201705
Anti-Rat CD45RA	Biolegend	Cat#202315
Anti-Rat CD11b	Biolegend	Cat#301306
Anti-Rat CD43	Biolegend	Cat#202810
Anti-Rat CD86	Biolegend	Cat#551396
Antibodies for Immunofluorescence and Western blot		
Anti-Rat PPARγ	Abcam	Cat#16643-1-AP
Anti-Rat CD68	Bio-Rad	Cat#MCA341GA
Alexa Fluor 488 Goat anti-Rabbit IgG	Invitrogen	Cat#A-11034
Alexa Fluor 555 Goat anti-Rabbit IgG	Invitrogen	Cat#A-21428
GAPDH Rabbit mAb	CST	Cat# 5174S

Supplementary Figures:

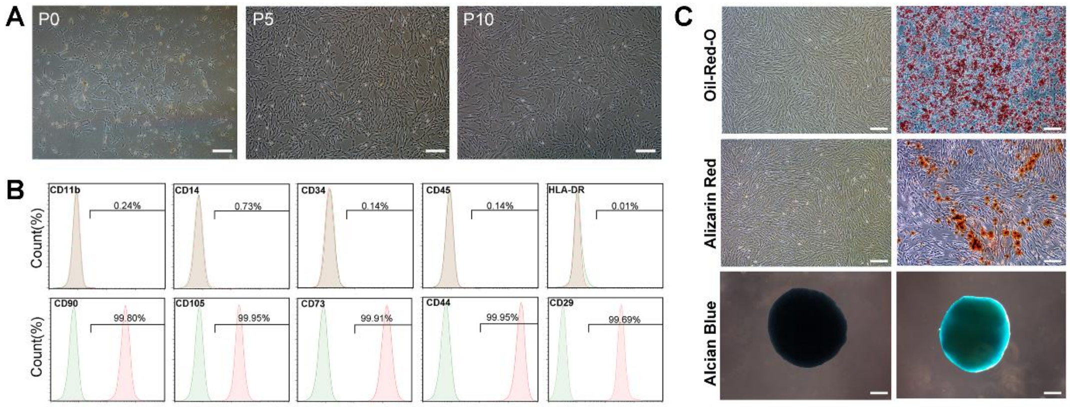

FigureS1.Culture and identification of hUCMSCs

(A) Morphological characteristics of hUCMSCs (Bar=100μm).

(B) Flow cytometry analysis of surface markers of hUCMSCs.

(C) Identification of multi-differentiation potential of hUCMSCs

(Bar=100μm).

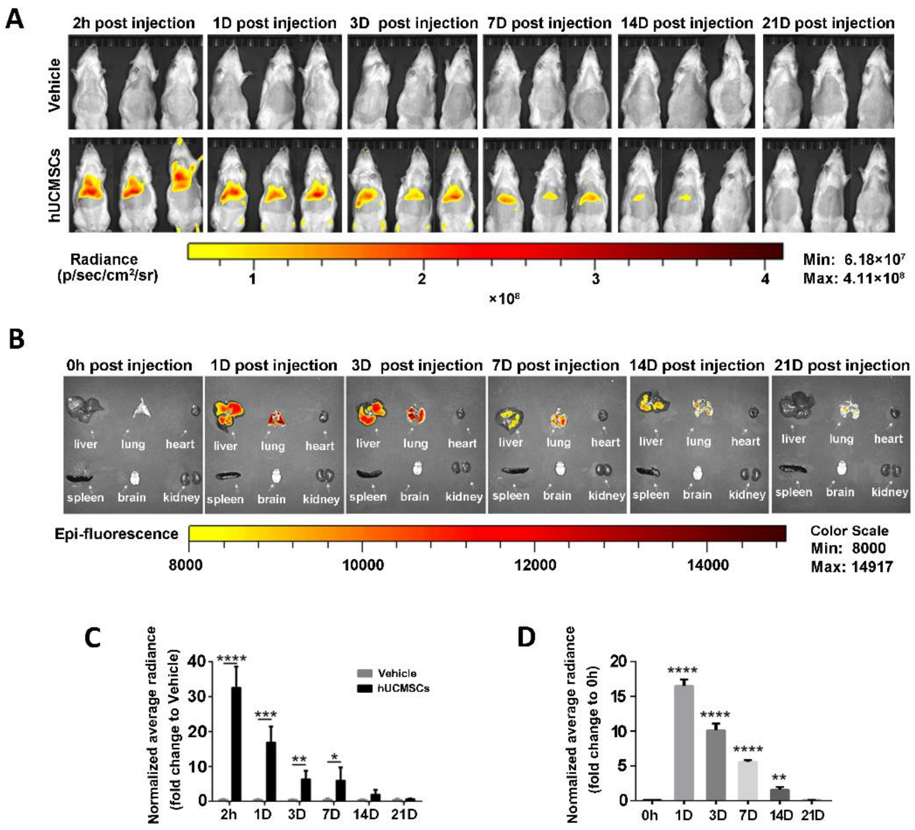

FigureS2. The biodistribution of hUCMSCs in DLC rats

(A) Representative IVIS images of 2h, 1D, 3D, 7D, 14D, and 21D post-injection of DiR-labeled hUCMSCs (n=3).

(B) Representative IVIS images of six organs collected at 2h, 1D, 3D, 7D, 14D, and 21D following i.v. transfusion of DiR-labeled hUCMSCs (n=3).

(C) Fluorescence intensity statistics analysis corresponding to Figure A (mean±SEM. n=3).

(D) Fluorescence intensity statistics analysis of the liver at different related-time points (mean±SEM. n=3).

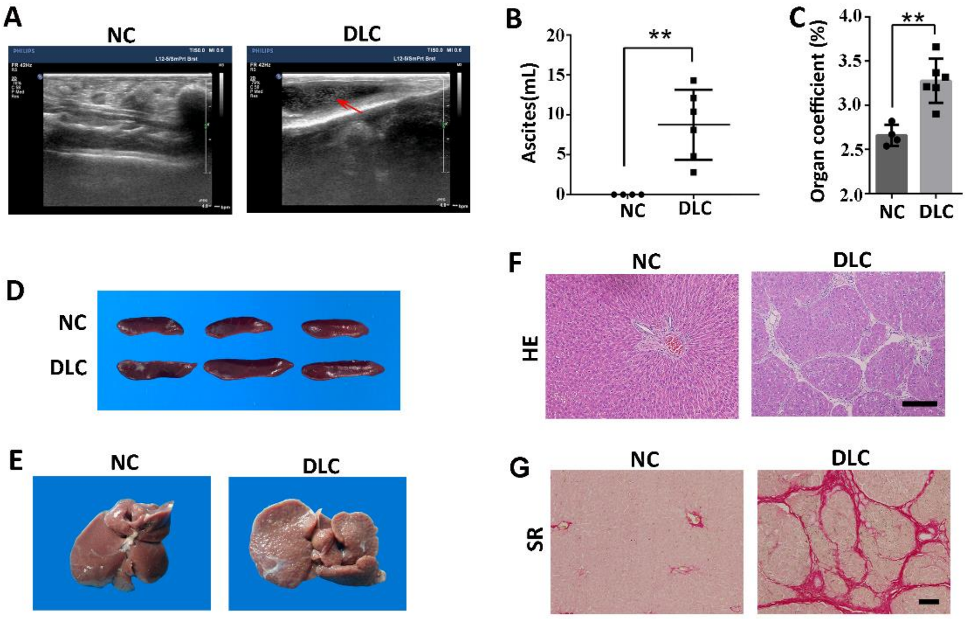

FigureS3. Typical characteristics of decompensated liver cirrhosis rats

(A) Abdominal ultrasound and the red arrow indicate ascites.

(B) Quantification analysis of the ascites (mean±SEM., NC group, n=4; DLC group, n=6).

(C) The ratio of liver weight to body weight in rats (mean±SEM., NC group, n=4; DLC group, n=6).

(D) Representative macroscopic appearance of the spleen.

(E) Representative macroscopic appearance of the liver.

(F) Hematoxylin and eosin (H&E) staining of liver sections (Bar=100μm).

(G) Sirius red (SR)staining of liver sections (Bar=100μm).

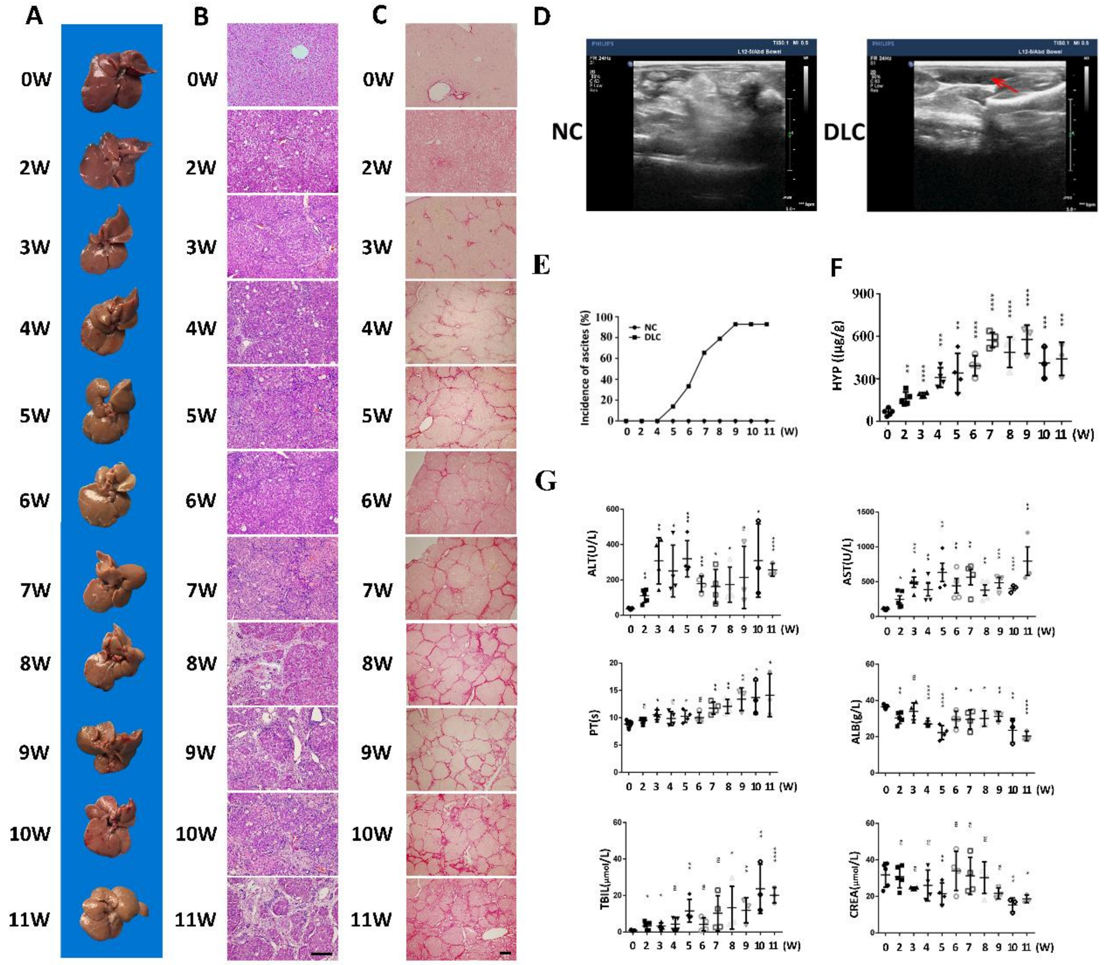

FigureS4. Changes in liver structure and function in DLC rats during the disease process

(A) Representative macroscopic appearance of the liver.

(B) Hematoxylin and eosin (H&E) staining of liver sections (Bar=100μm).

(C) Sirius red (SR)staining of liver sections (Bar=100μm).

(D)Development of ascites in DLC model rats.

(E) Incidence of ascites in DLC rats (mean±SEM, n=5).

(F)Liver hydroxyproline acid content in DLC rats (mean±SEM, n=4).

(G) Serum AST, ALT, PT, ALB, TBIL, and CREA levels in indicated rats (mean±SEM, n=4).

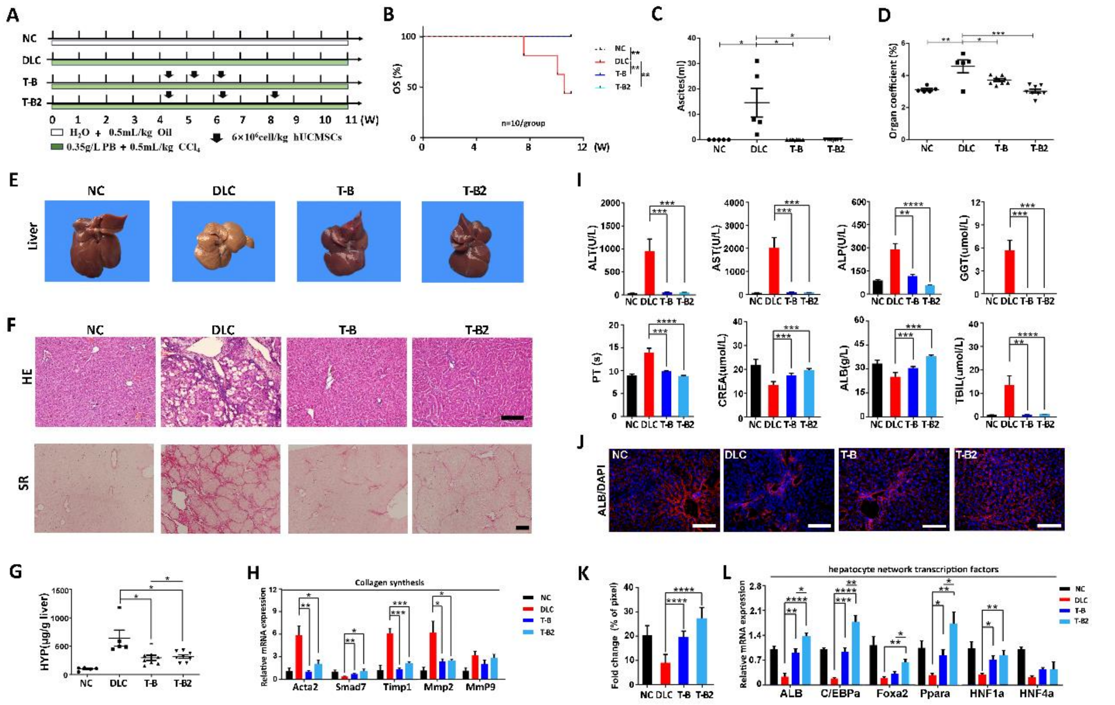

FigureS5. Therapeutic effects of infusion interval of hUCMSCs treatment on DLC rats.

(A) Diagram of weekly and biweekly hUCMSCs infusion regimens on DLC rats.

(B) The survival of the rats was recorded from week six until the end of week 11(n=10).

(C) Statistics of ascites production (mean±SEM, NC, and DLC group, n=5; T-B and T-B2 group, n=8).

(D) Ratio of the liver weight to the body weight in rats at week 11 of modeling (mean±SEM, NC and DLC group, n=5; T-B and T-B2 group, n=8).

(E) The representative images of the liver appearance.

(F) Hematoxylin and eosin (H&E) and Sirius red (SR)staining of liver sections.

(G) Hydroxyproline content of liver tissues (mean±SEM, NC, and DLC group, n=5; T-B and T-B2 group, n=8).

(H) The expression levels of collagen synthesis-related genes in liver tissues (mean±SEM, NC, and DLC group, n=5; T-B and T-B2 group, n=8).

(I) Serum levels of key enzymes related to liver function (mean±SEM, NC, and DLC group, n=5; T-B and T-B2 group, n=8).

(J) Representative liver sections from each group were stained with fluorescent ALB (red fluorescence).

(K) Fluorescence statistics of ALB positive cells (mean±SEM, NC, and DLC group, n=5; T-B and T-B2 group, n=8).

(L) The expression levels of hepatocyte network transcription factors-related genes in liver tissues (mean±SEM, NC, and DLC group, n=5; T-B and T-B2 group, n=8).

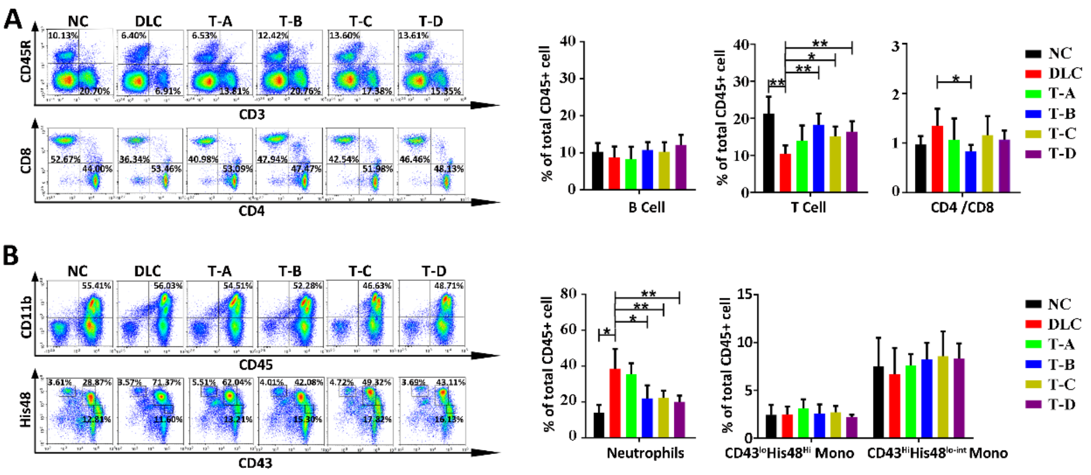

FigureS6. Changes in liver structure and function in DLC rats during the disease process

(A) Flow cytometry examination of the proportions of T cells and B cells in liver

tissues of the NC group, DLC group, and different hUCMSCs groups(n=5).

(B) The proportion of neutrophils, monocytes, and their subtypes in liver tissues of the NC group, DLC group, and different hUCMSCs groups were detected by flow cytometry(n=5).

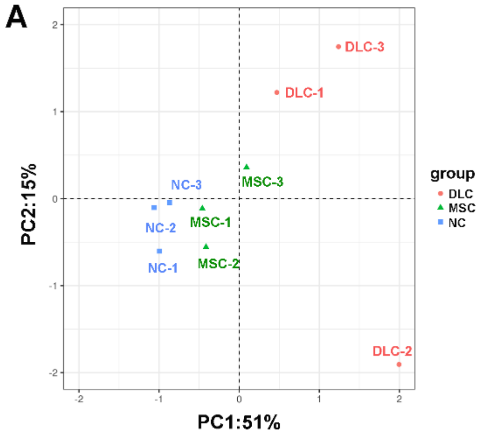

FigureS7. Principal component analysis of liver tissue samples

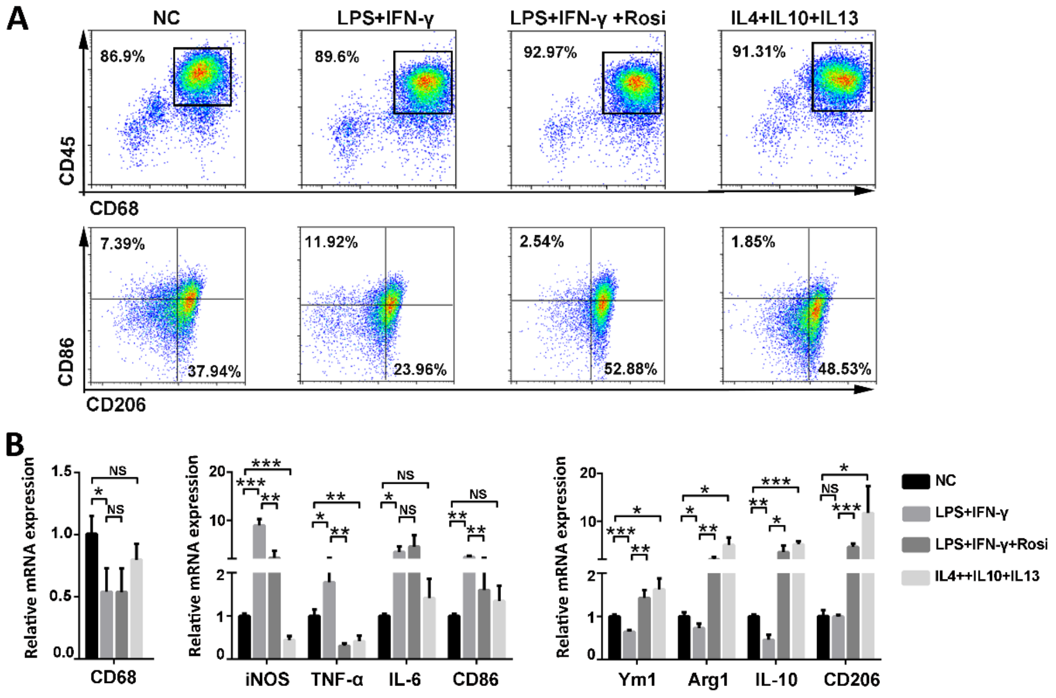

FigureS8. The effect of PPARγ agonists rosiglitazone on the phenotype of macrophages

(A) Flow cytometry analysis of the rosiglitazone effect on the polarization of

macrophages.

(B) RT-qPCR analysis of the rosiglitazone effect on the expression of M1-related and M2-related genes in macrophages (mean±SEM, n=3).

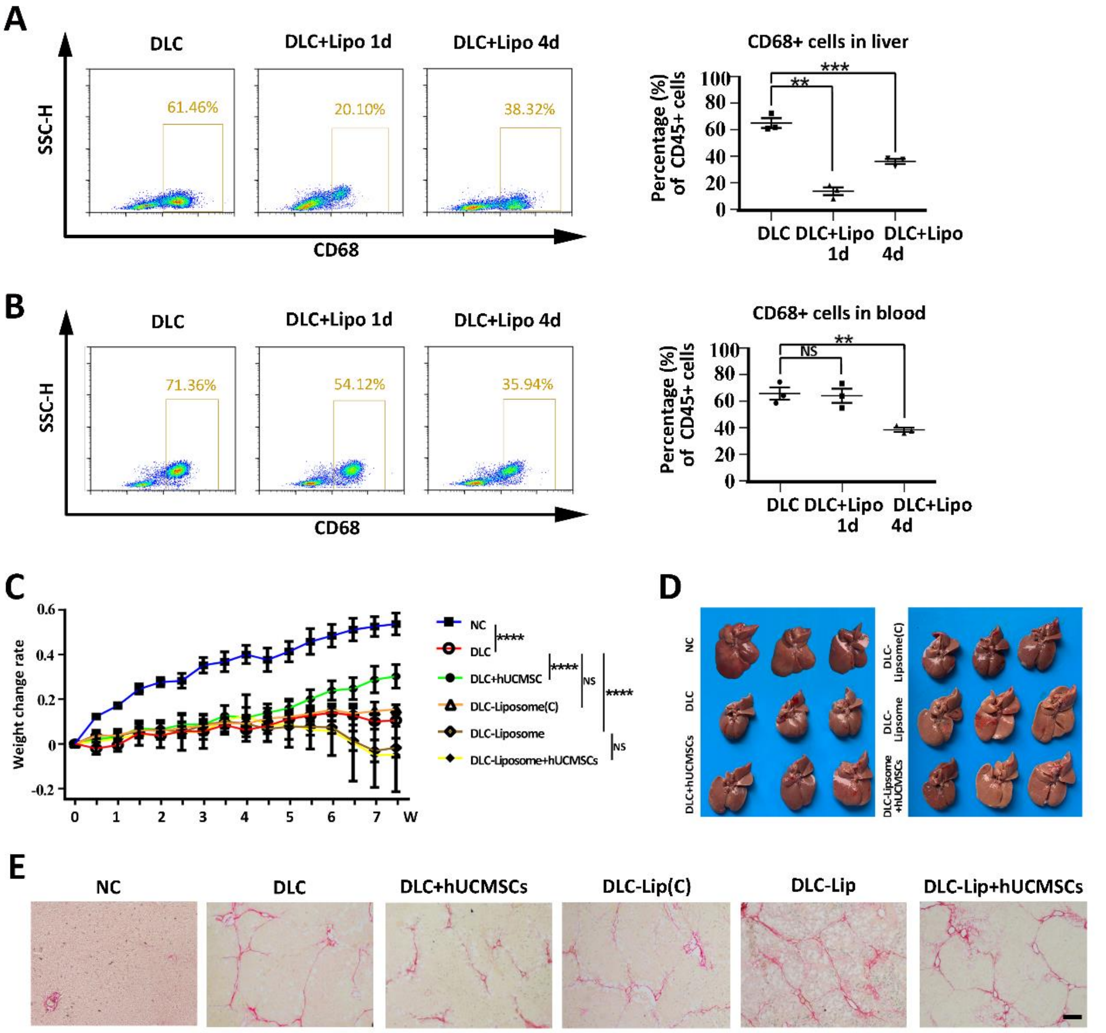

FigureS9. The efficiency of single depletion in liver macrophages in DLC rats and the effects of hUCMSCs treatment

(A) Flow cytometry analysis of CD68 positive cells in DLC rats' liver at day one and day four after a single injection of chlorophosphate liposomes (mean±SEM, n=3).

(B) Flow cytometry analysis of CD68 positive cells in the blood of DLC rats at day one and day four after a single injection of chlorophosphate liposomes (mean±SEM, n=3).

(C) Quantification of body weight in different groups for seven weeks (mean±SEM, NC and DLC group, n=5; DLC+hUCMSCs and DLC-Lip(C) group, n=4; DLC-Lip and DLC-Lip+hUCMSCs group, n=3).

(D) The general appearance of the livers of the six groups of rats at seven weeks.

(E) Sirius red (SR)staining of liver sections (Bar=100μm).

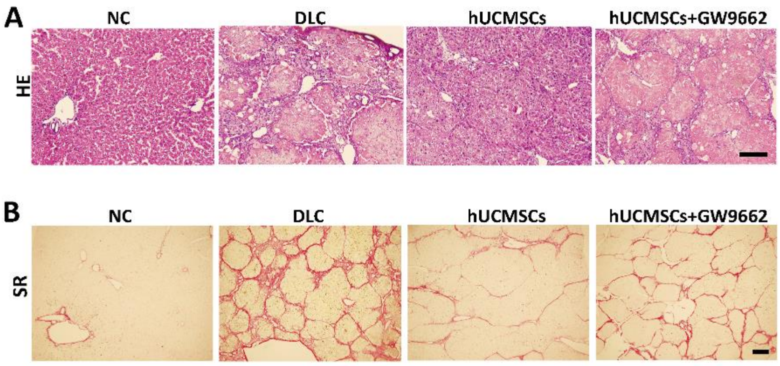

FigureS10. PPARγ inhibition attenuates the therapeutic effect of hUCMSCs on DLC rats

(A) Hematoxylin and eosin (H&E) staining of liver sections (Bar=100μm).

(B) Sirius red (SR)staining of liver sections (Bar=100μm).
