# Supplementary material for: PPARγ-dependent hepatic macrophage switching acts as a central hub for hUCMSC-mediated alleviation of decompensated liver cirrhosis in rats
Source: Stem Cell Res Ther. 2023 Jul 27;14:184. doi: 10.1186/s13287-023-03416-2 (PMC10375757; doi:10.1186/s13287-023-03416-2)
Supplement: Supplementary file 2 — Additional file 2: Original blot images of Figure 3E. [file 13287_2023_3416_MOESM2_ESM.docx]

**SF1-1. Original western blot gels of Fig.3E for protein expression levels of PPARγ in different rats’ liver groups.**

**180kDa**


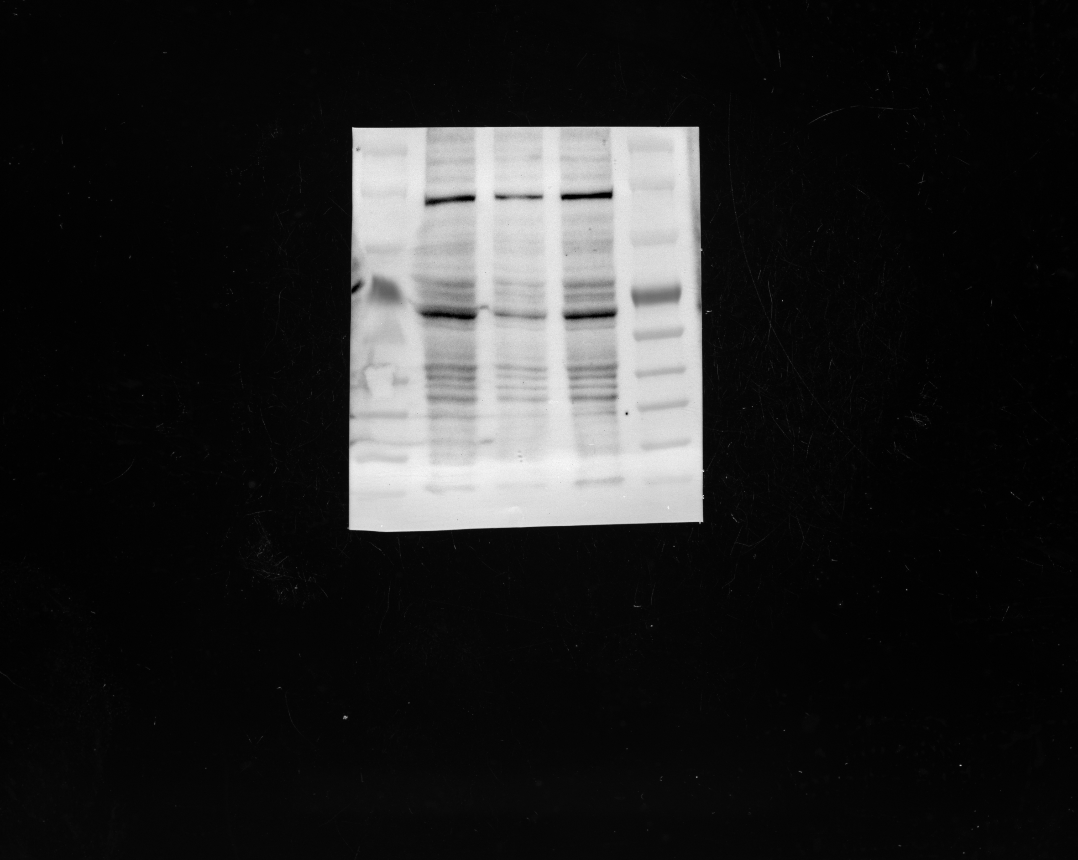


**130kDa**

**100kDa**

**PPARγ**

**70kDa**

**55kDa**

**40kDa**

**35kDa**

**15kDa**

**25kDa**


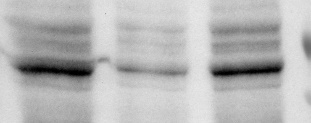

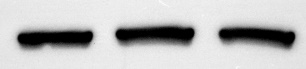


**PPARγ**

**GAPDH**

**NC**

**hUCMSCs**

**DLC**


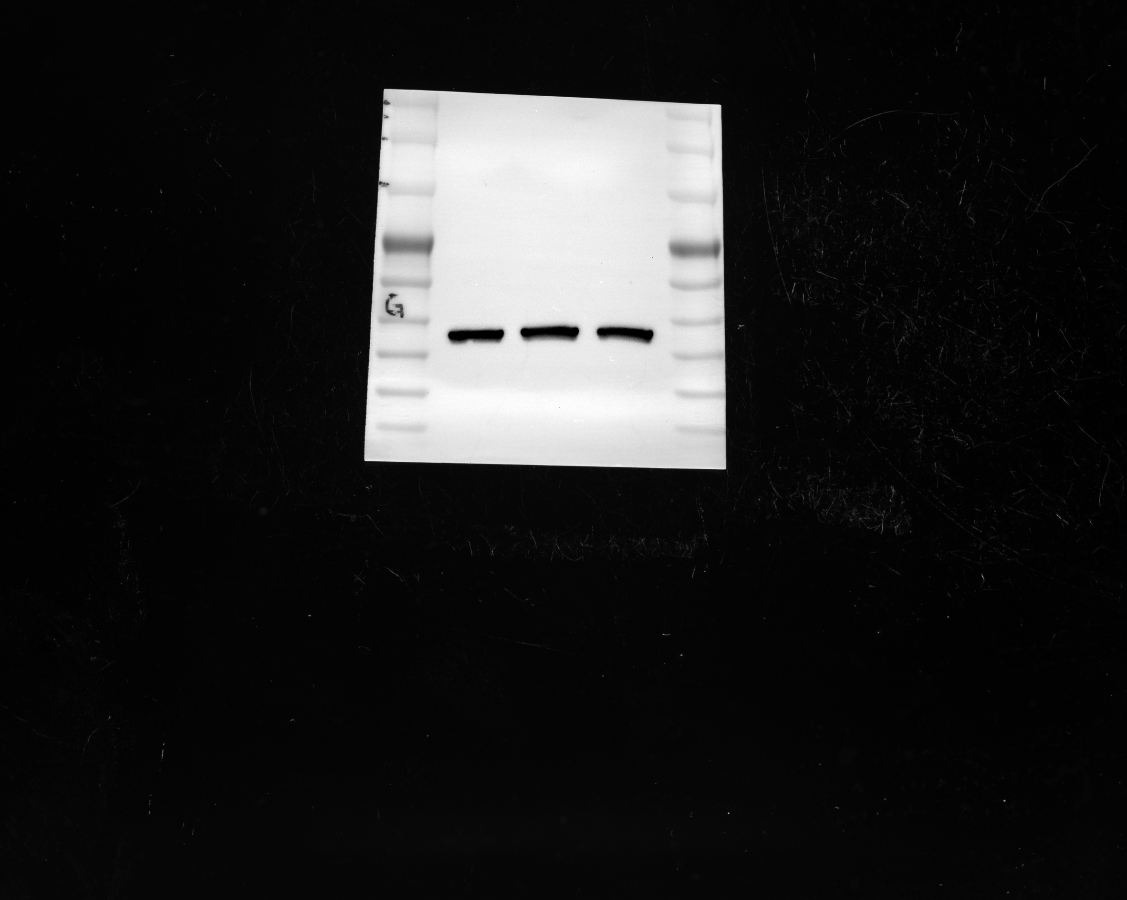
­

**180kDa**

**130kDa**

**100kDa**

**70kDa**

**55kDa**

**GAPDH**

**35kDa**

**40kDa**

**25kDa**

**15kDa**

­

**SF1-2. Original western blot gels of repeated experiments of Fig.3E.**


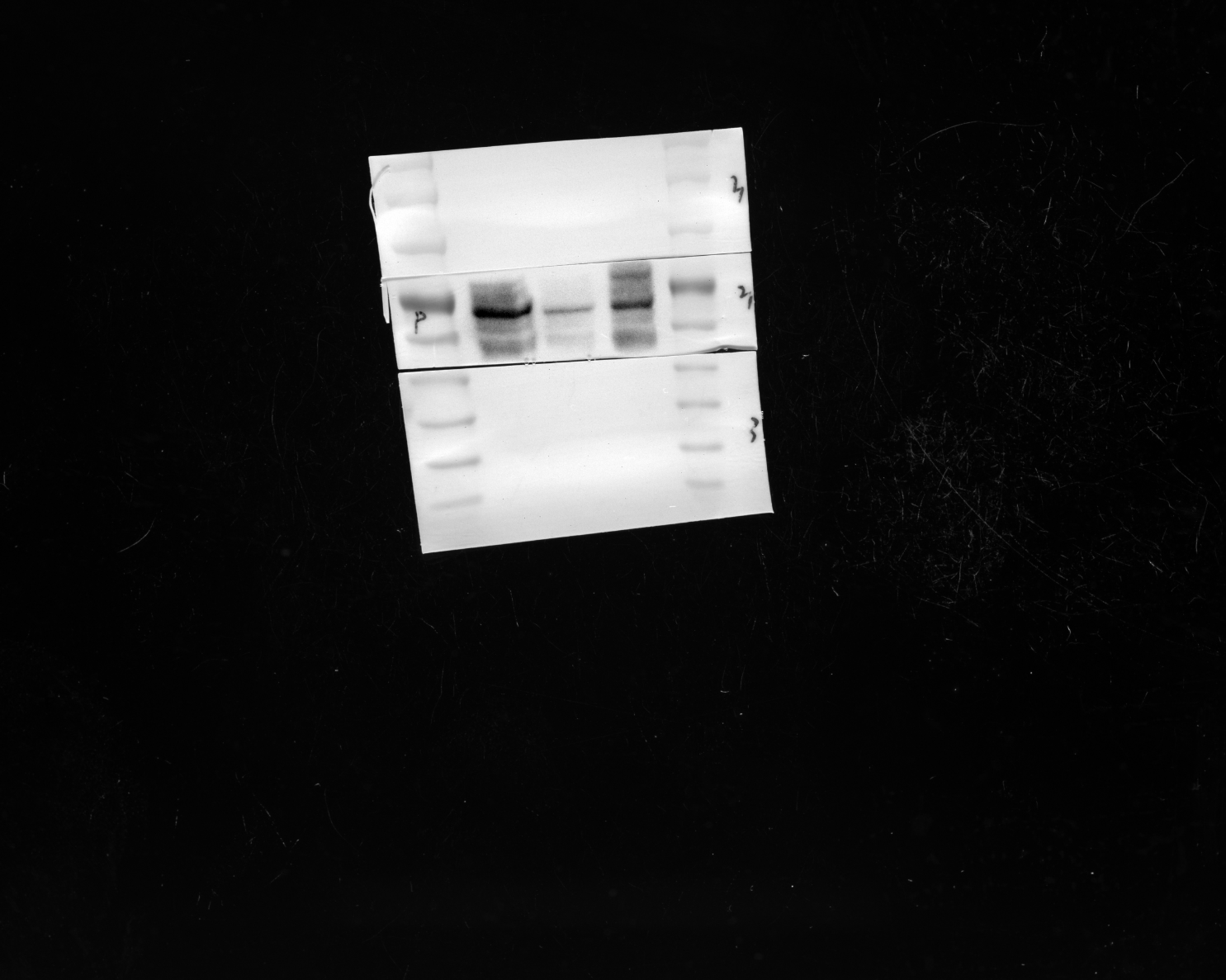


**180kDa**

**130kDa**

**100kDa**

**PPARγ**

**70kDa**

**55kDa**

**40kDa**

**35kDa**

**25kDa**

**15kDa**


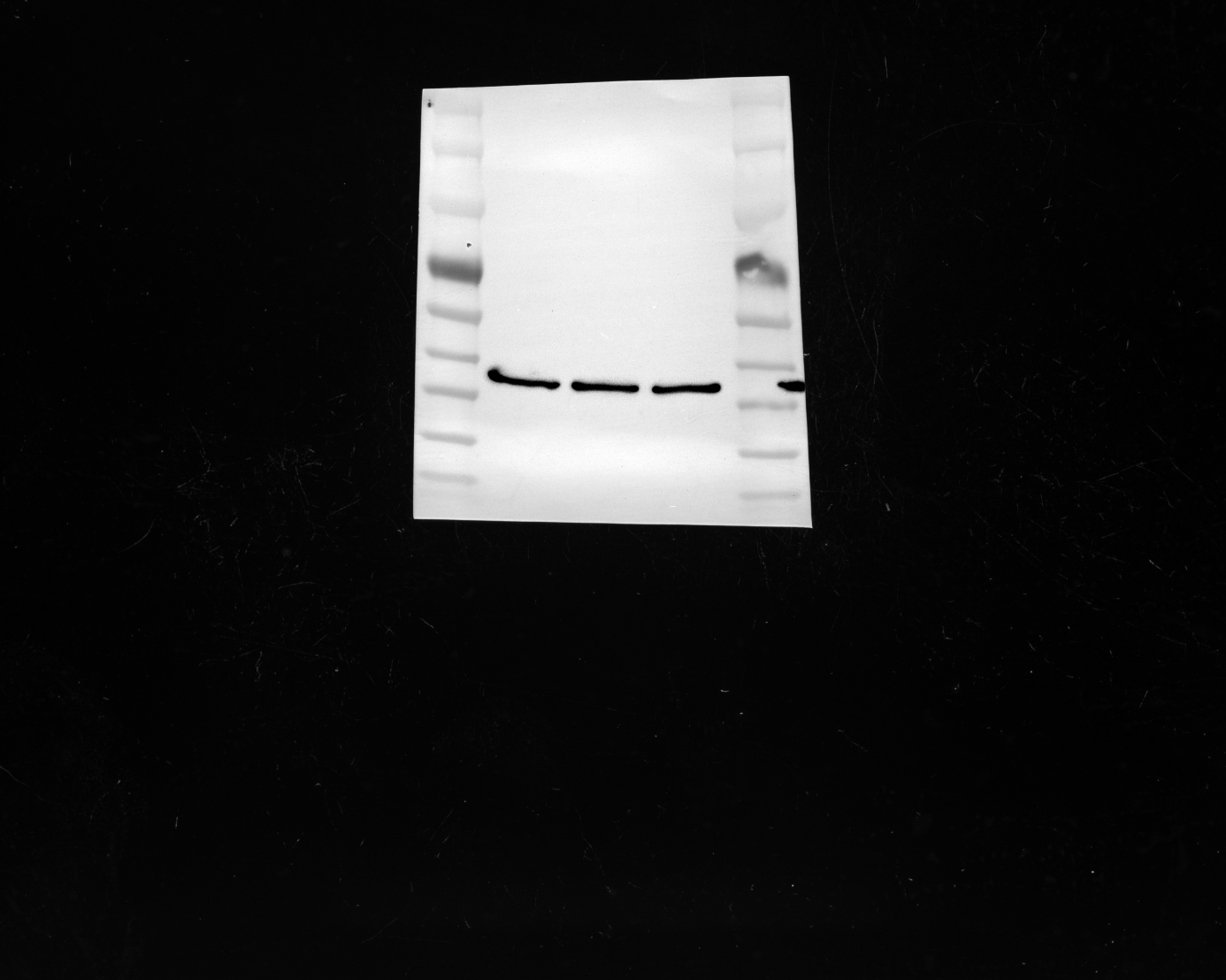


**180kDa**

**130kDa**

**100kDa**

**70kDa**

**55kDa**

**GAPDH**

**40kDa**

**35kDa**

**25kDa**

**15kDa**

**SF1-3. Original western blot gels of repeated experiments of Fig.3E.**


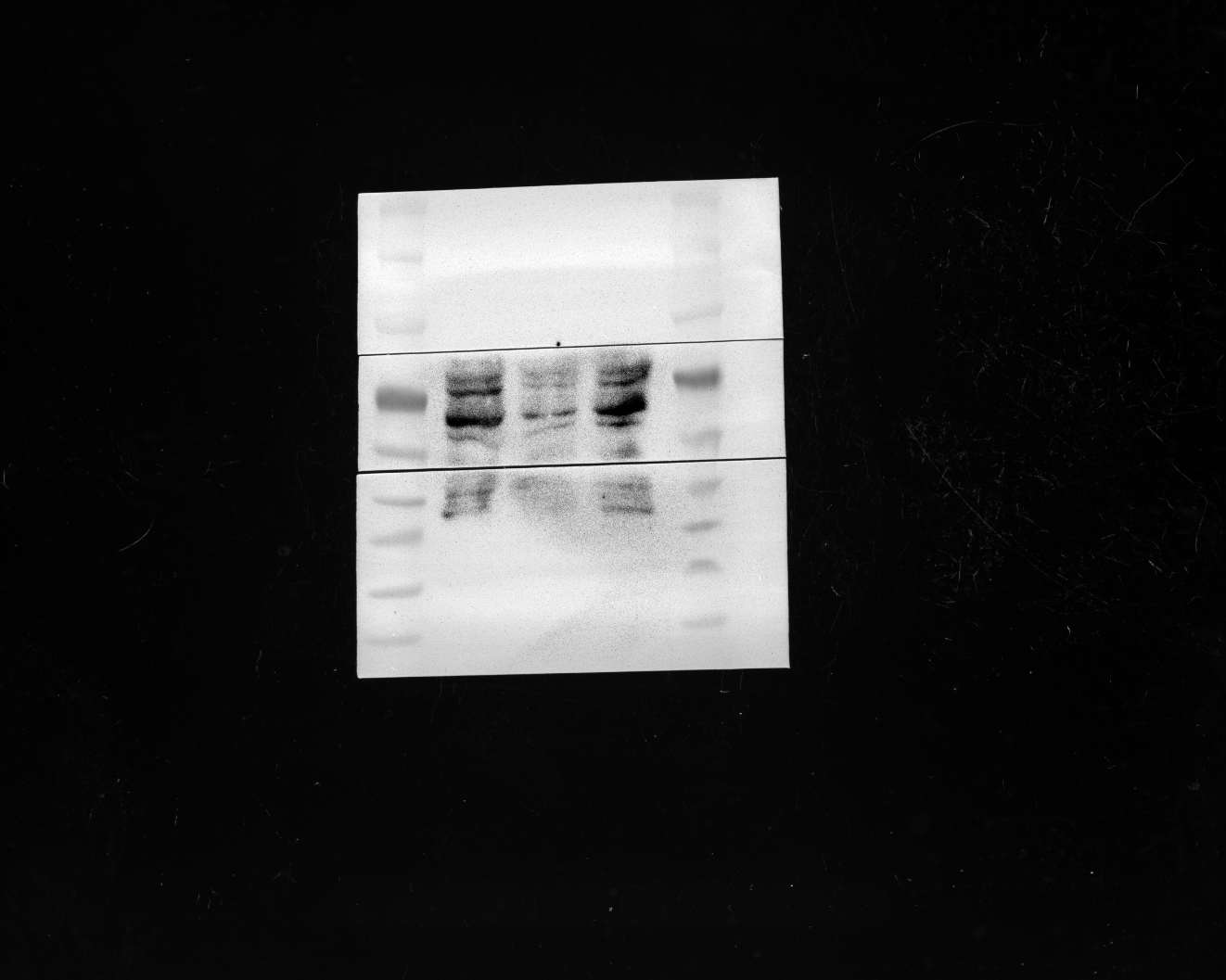


**180kDa**

**130kDa**

**100kDa**

**PPARγ**

**70kDa**

­­

**55kDa**

**40kDa**

**35kDa**

**25kDa**

**15kDa**


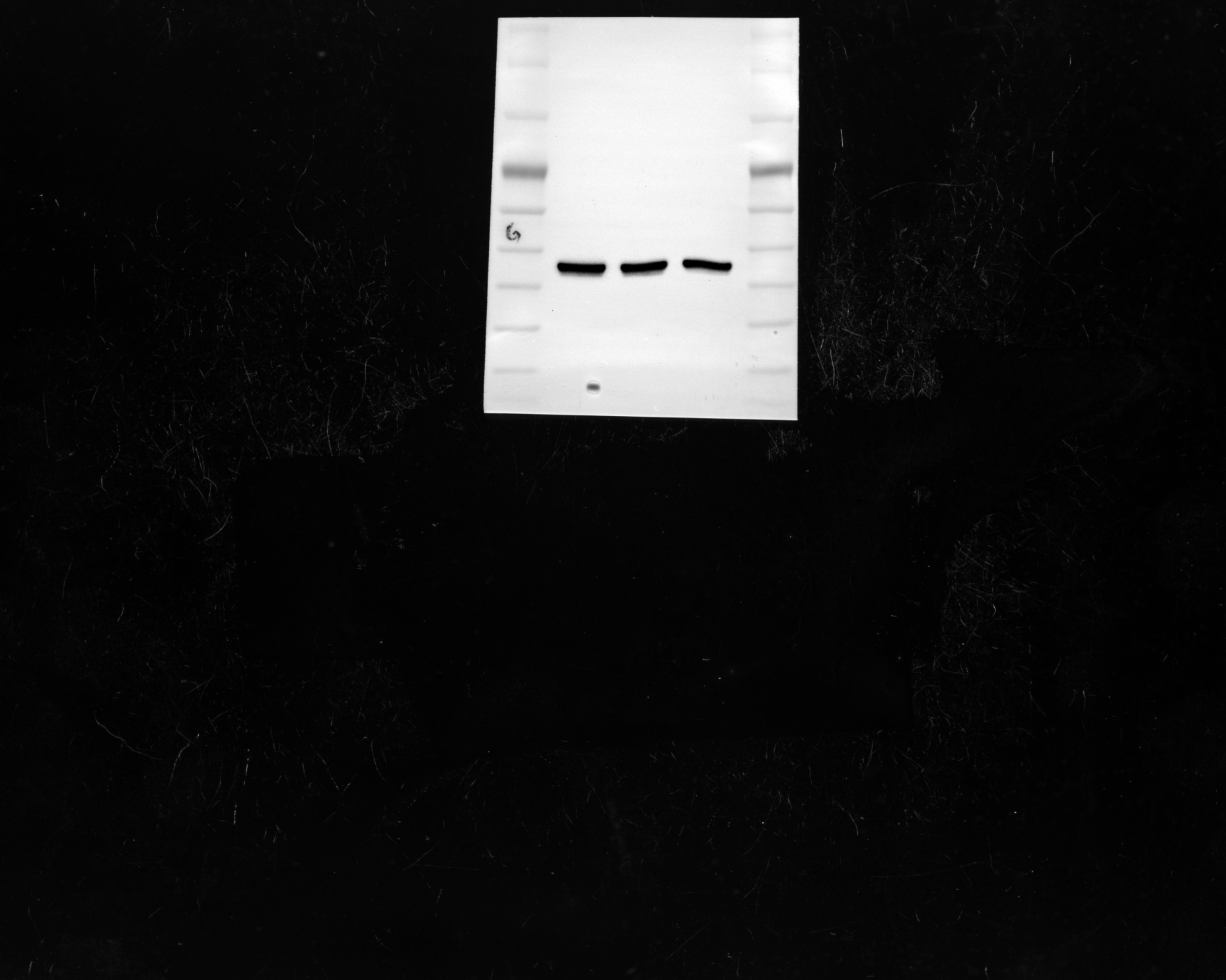


**180kDa**

**130kDa**

**100kDa**

**70kDa**

**55kDa**

**40kDa**

**GAPDH**

**35kDa**

**25kDa**

**15kDa**

**SF1-4. Original western blot gels of repeated experiments of Fig.3E.**


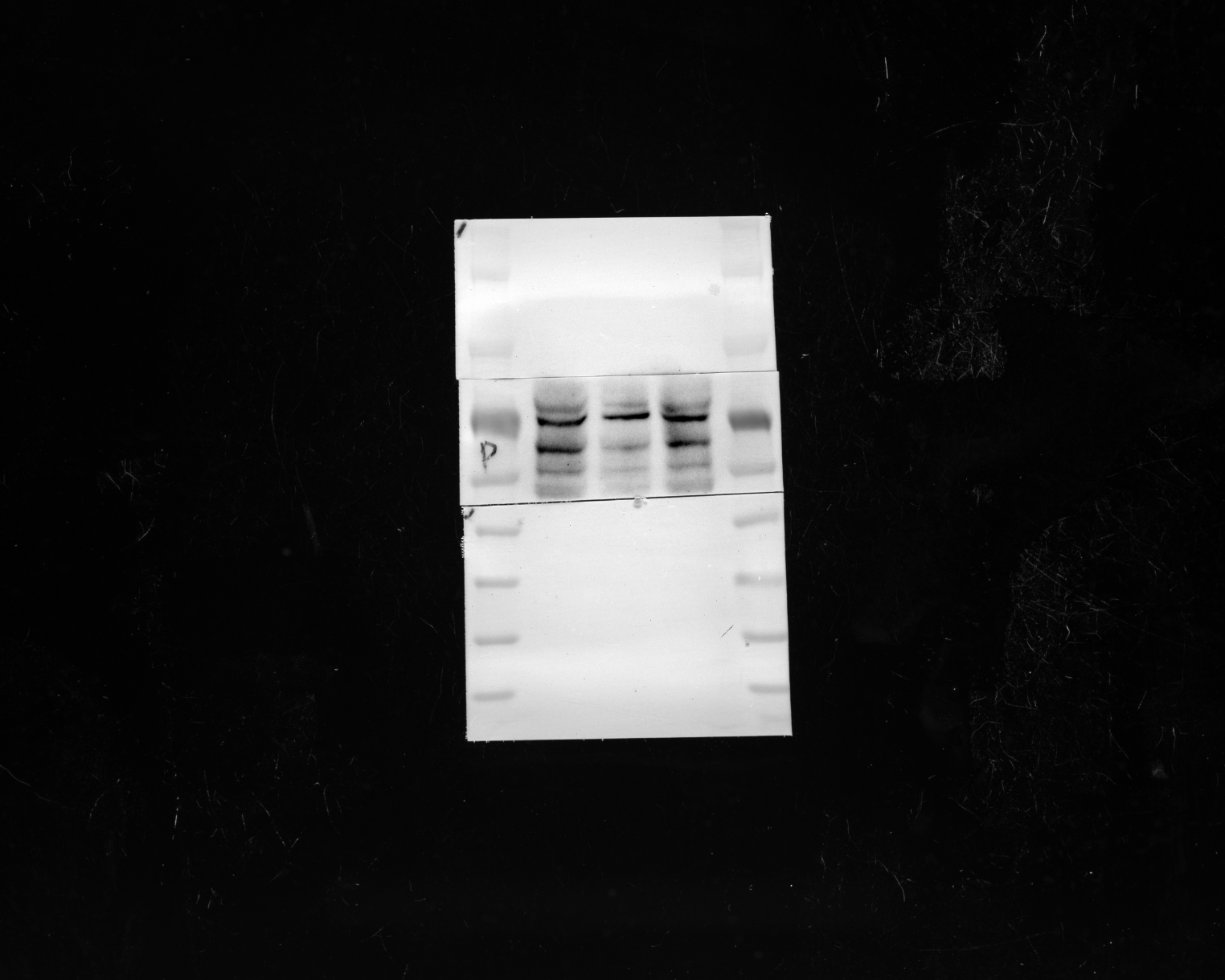


**180kDa**

**130kDa**

**100kDa**

**PPARγ**

**70kDa**

**55kDa**

**40kDa**

**35kDa**

**25kDa**

**15kDa**


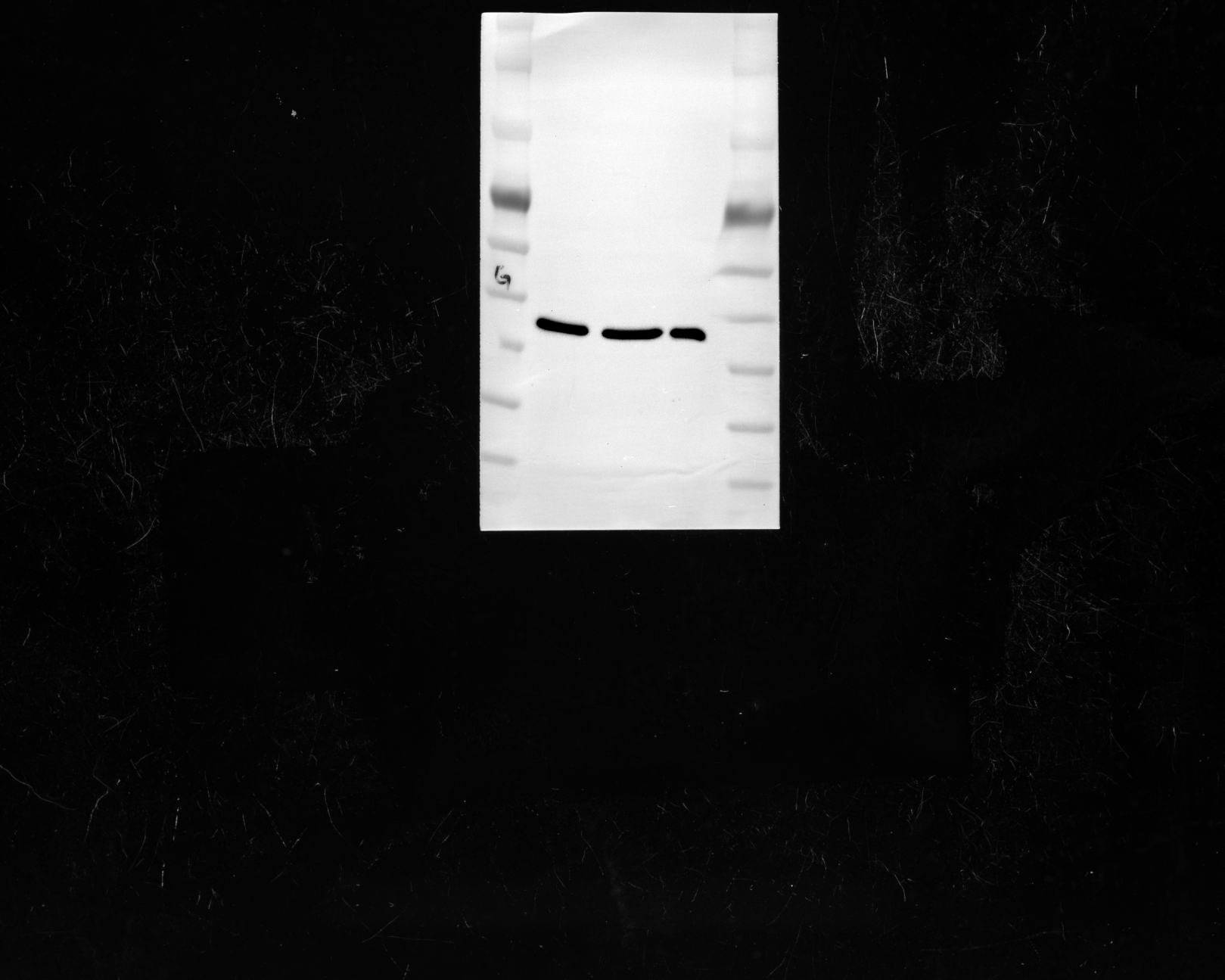


**100kDa**

**GAPDH**

**180kDa**

**130kDa**

**70kDa**

**55kDa**

**40kDa**

**35kDa**

**25kDa**

**15kDa**
