# Supplementary material for: PPARγ-dependent hepatic macrophage switching acts as a central hub for hUCMSC-mediated alleviation of decompensated liver cirrhosis in rats
Source: Stem Cell Res Ther. 2023 Jul 27;14:184. doi: 10.1186/s13287-023-03416-2 (PMC10375757; doi:10.1186/s13287-023-03416-2)
Supplement: Supplementary file 3 — Additional file 3: Original blot images of Figure 5C. [file 13287_2023_3416_MOESM3_ESM.docx]

**SF2-1. Original western blot gels of Fig.5C for protein expression levels of PPARγ in macrophages.**

­


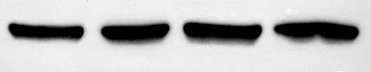

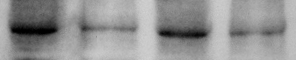


**LPS+IFNγ+**

**hUCMSCs+GW9662**

**LPS+IFNγ+hUCMSCs**

**LPS+IFNγ**

**NC**

**GAPDH**

**PPARγ**


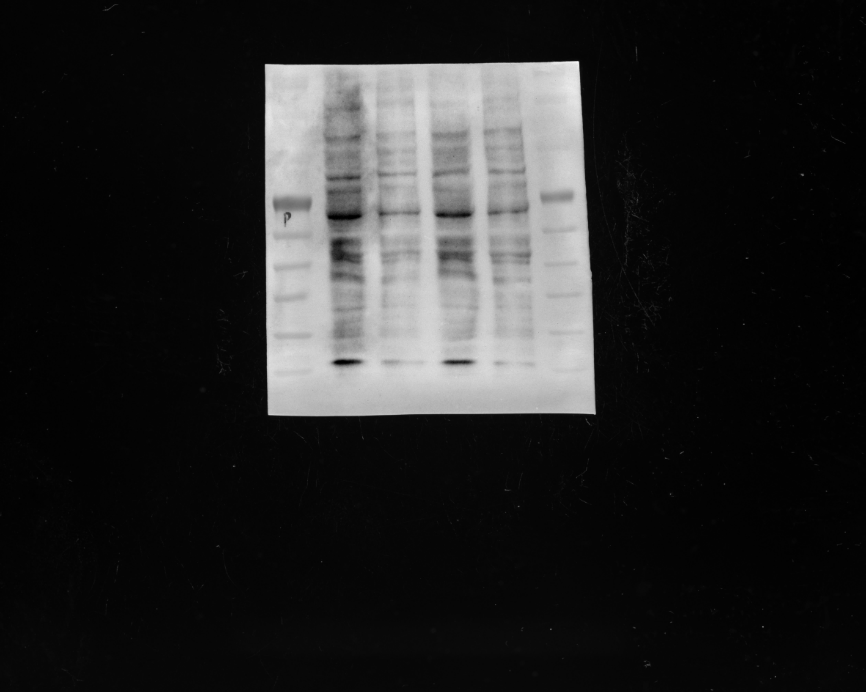


**100kDa**

**130kDa**

**180kDa**

**PPARγ**

**55kDa**

**70kDa**

**25kDa**

**35kDa**

**40kDa**

**15kDa**

**
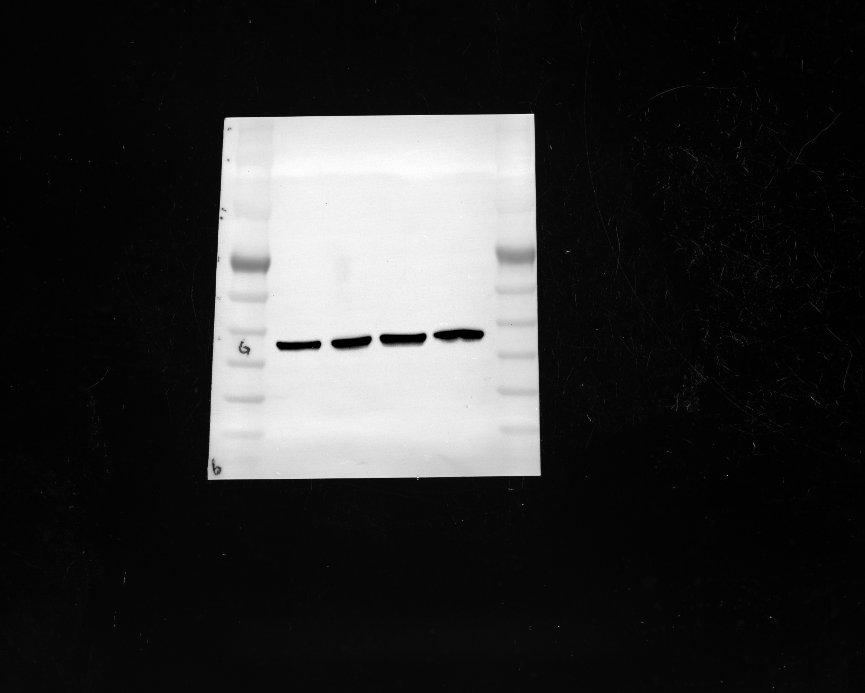
**

**130kDa**

**180kDa**

**70kDa**

**100kDa**

**GAPDH**

**55kDa**

**35kDa**

**40kDa**

**15kDa**

**25kDa**

**SF2-2. Original western blot gels of repeated experiments of Fig.5E.**

**
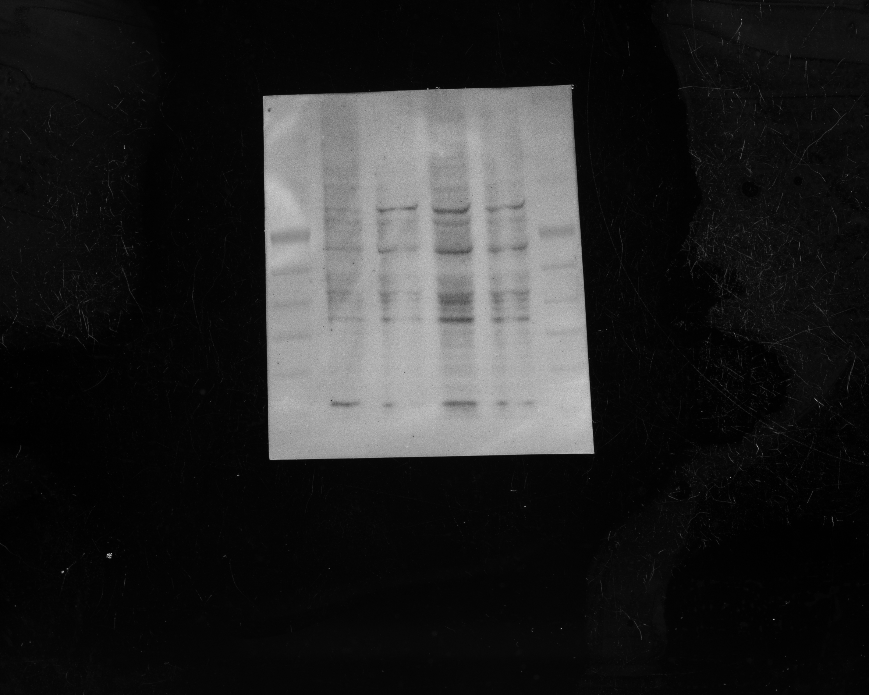
**

**130kDa**

**180kDa**

**100kDa**

**40kDa**

**55kDa**

**25kDa**

**PPARγ**

**70kDa**

**35kDa**

**15kDa**

**
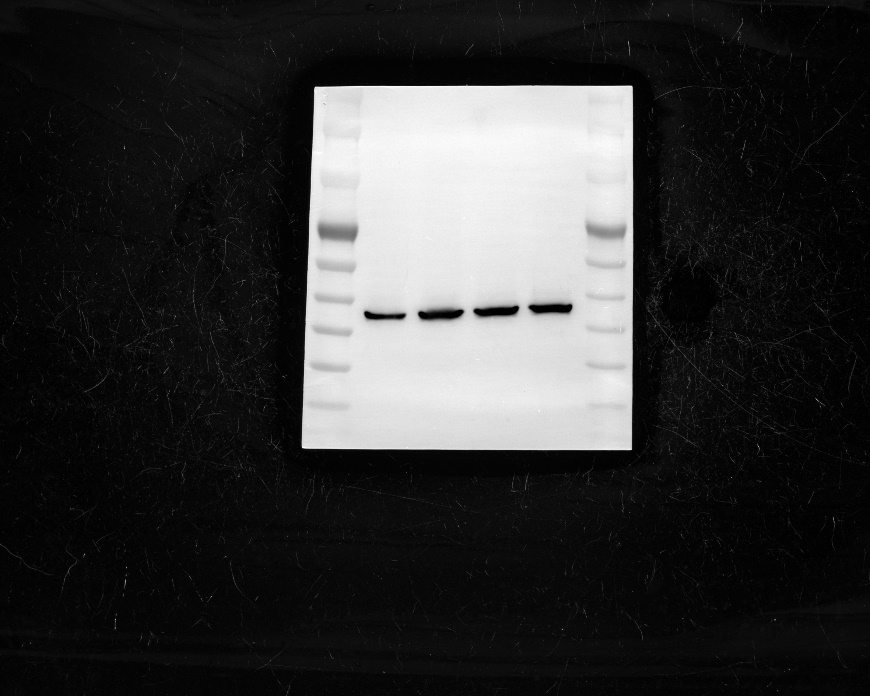
**

**100kDa**

**130kDa**

**180kDa**

**55kDa**

**70kDa**

**GAPDH**

**25kDa**

**35kDa**

**40kDa**

**15kDa**

**SF2-3. Original western blot gels of repeated experiments of Fig.5C.**

**
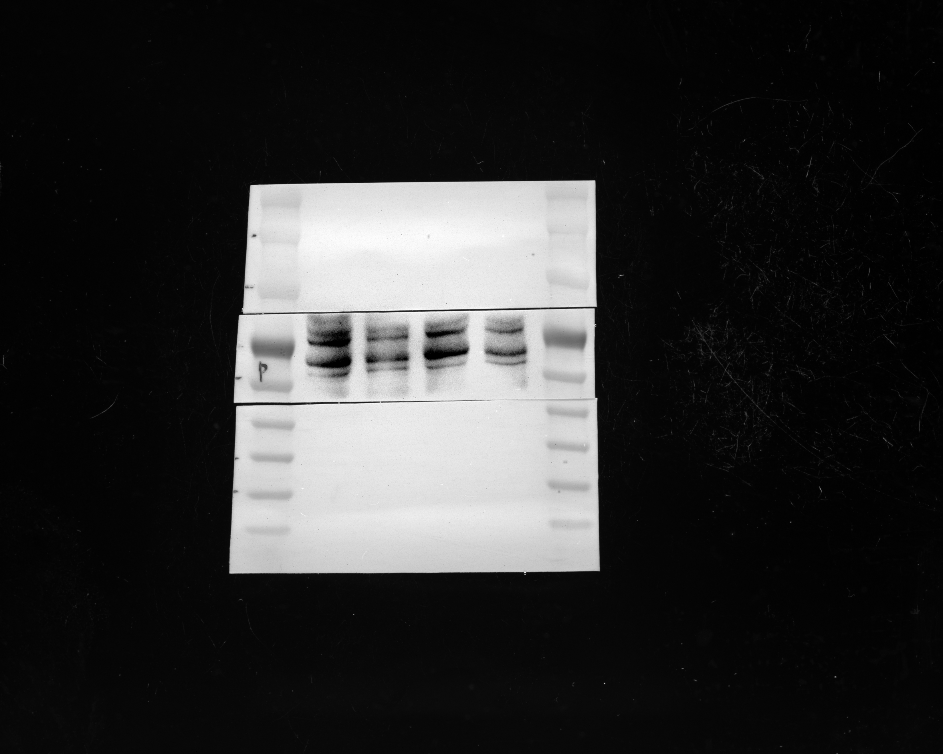
**

**130kDa**

**180kDa**

**100kDa**

**PPARγ**

**40kDa**

**55kDa**

**70kDa**

**15kDa**

**25kDa**

**35kDa**

**180kDa**

**
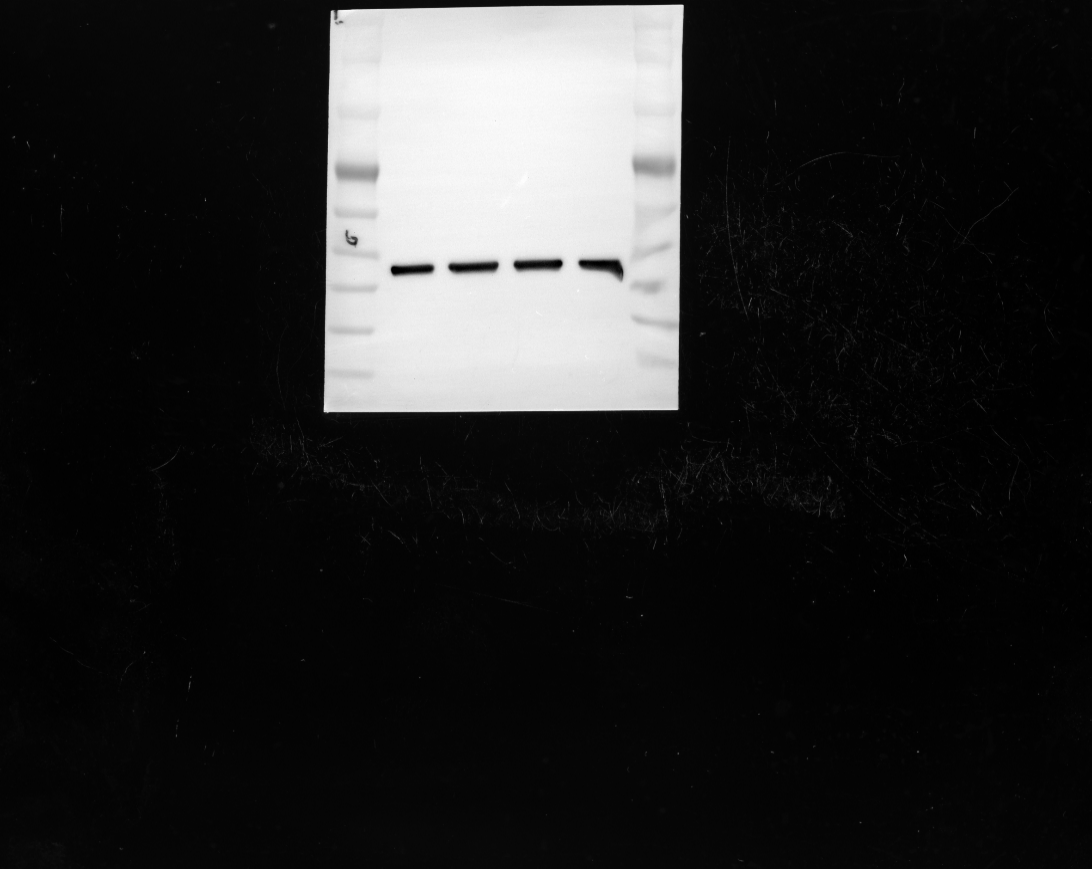
**

**GAPDH**

**100kDa**

**15kDa**

**35kDa**

**40kDa**

**25kDa**

**55kDa**

**70kDa**

**130kDa**
